# A showcase study on personalized in silico drug response prediction based on the genetic landscape of muscle invasive bladder cancer

**DOI:** 10.1038/s41598-021-85151-3

**Published:** 2021-03-12

**Authors:** Friedemann Krentel, Franziska Singer, María Lourdes Rosano-Gonzalez, Ewan A. Gibb, Yang Liu, Elai Davicioni, Nicola Keller, Daniel J. Stekhoven, Marianna Kruithof-de Julio, Roland Seiler

**Affiliations:** 1 Department of Urology, University of Bern, 3010 Bern, Switzerland; 2 NEXUS Personalized Health Technologies, ETH Zurich, Zurich, Switzerland; 3 SIB Swiss Institute of Bioinformatics, Lausanne, Switzerland; 4 GenomeDx Biosciences, Vancouver, Canada; 5 University of Basel, Basel, Switzerland; 6 Department for BioMedical Research, Urology Research Laboratory, University of Bern, Bern, Switzerland; 7 Translational Organoid Research, Department for BioMedical Research, University of Bern, Bern, Switzerland; 8 Bern Center for Precision Medicine, University of Bern, Bern University Hospital, Bern, Switzerland

**Keywords:** Bladder cancer, Cancer genetics, Targeted therapies

## Abstract

Improved and cheaper molecular diagnostics allow the shift from “one size fits all” therapies to personalised treatments targeting the individual tumor. However, the wealth of potential targets based on comprehensive sequencing remains a yet unsolved challenge that prevents its routine use in clinical practice. Thus, we designed a workflow that selects the most promising treatment targets based on multi-omics sequencing and in silico drug prediction. In this study we demonstrate the workflow with focus on bladder cancer (BLCA), as there are, to date, no reliable diagnostics available to predict the potential benefit of a therapeutic approach. Within the TCGA-BLCA cohort, our workflow identified a panel of 21 genes and 72 drugs that suggested personalized treatment for 95% of patients—including five genes not yet reported as prognostic markers for clinical testing in BLCA. The automated predictions were complemented by manually curated data, thus allowing for accurate sensitivity- or resistance-directed drug response predictions. We discuss potential improvements of drug-gene interaction databases on the basis of pitfalls that were identified during manual curation.

## Introduction

The rapid advance of technologies for the molecular profiling of tumors led to the discovery of novel treatments targeting the individual tumor. This allowed a shift from “one size fits all” to a more specific treatment in several cancer entities, such as breast, lung and colorectal cancer. However, a standardized method to identify potential targets from molecular data is still missing and prevents the implementation in clinical practice. In comparison to other cancer entities, in bladder cancer (BLCA) potential treatment targets are still relatively poorly investigated and individualized treatment concepts are far behind other cancer entities. Therefore, we aimed to design a workflow for an in silico drug prediction with an in-depth showcase study on muscle-invasive bladder cancer.

More than 3 decades of development in empiric systemic therapies have passed without remarkably changing the numbers of deaths caused by bladder cancer^1^. Recent advancements include immunotherapies in BLCA, which have brought the first significant progress in survival since platinum-based chemotherapy regimens were introduced in the mid 1980s^2^. However, in recent years the 5-year overall survival has only been improved by 5%—and the long-term effects of immunotherapies are still to be evaluated^3^.

BLCA is among the cancer types with the highest total mutational burden^4^. This molecular diversity of BLCA might be one of the major reasons for the low response rates of BLCA to empiric treatments. Moreover, about two thirds of BLCA are exposed to APOBEC mutagenesis, which increases the mutational diversity within each individual tumor during development. At the transcriptomic level, BLCA can be divided into molecular subtypes and clinical implications of these classifications have already been suggested^5^. Nevertheless, transcriptome-based classifications alone do not seem to be optimal for selecting the right therapeutic approach, as subtypes are still highly heterogeneous and do not adequately reflect the genomic individuality of each BLCA tumor. Improved and cheaper genomic sequencing techniques made routine use of molecular diagnostics affordable, which potentially allows the shift from “one size fits all” to personalized therapies.

To facilitate such a genomic marker-driven personalized therapy approach in BLCA, the first step is the identification of clinically relevant and actionable genomic alterations. Thus, in this study we used the TCGA-BLCA cohort as a starting point and aimed to determine potentially actionable genes and associated drugs based on the mutational landscape of muscle invasive bladder cancer. Moreover, we integrated transcriptomic data and existing evidence from other cancer entities to enrich the genomic-based information. The TCGA cohort based predictions and the developed workflow can now be used as a prognostic tool to further shift pretreatment diagnostics and decision making from an empiric towards an individualized marker-driven therapy strategy.

## Results

### Variant calling, quality control and variant filtering

**Figure 1:**
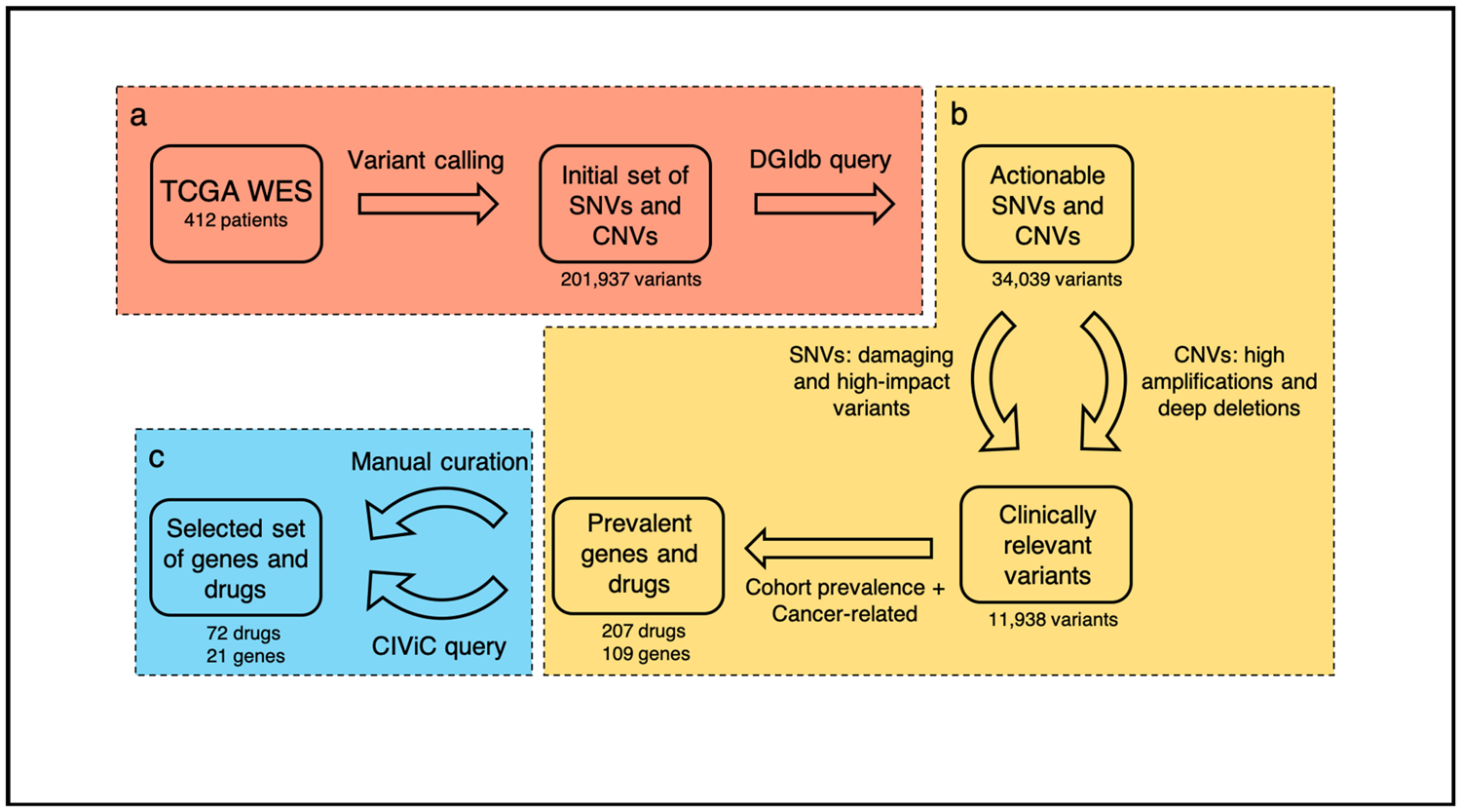
Workflow design—the diagram depicts the key steps of our in silico drug prediction. (**a**) After variant calling of whole-exome sequencing (WES) data from the TCGA bladder cancer cohort, a query was performed on the “Drug Gene Interaction database” (DGIdb) to identify targetable genomic alterations amongst the discovered mutations. (**b**) During the subsequent fully automated filtering phase, further selection criteria were applied. (**c**) In the semi-automated filtering phase manual curation complemented the automated query of the expert curated CIViC (“Clinical Interpretation of Variants in Cancer”) database, resulting in the final set of genes and drugs. (Microsoft PowerPoint; version 16.44).

We first performed variant calling from whole-exome sequencing (WES) data from the TCGA-BLCA cohort^6^. The derived single nucleotide variants (SNVs) and copy number variations (CNVs) served as a base for a query in “The Drug Gene Interaction Database” (DGIdb)^7^ to identify variants with non-curated* drug gene interaction (DGI) information (* = all DGIs not yet curated by manual curation or not derived from an expert curated DGI database will hereafter be called “non-curated” to highlight their level of evidence). Intending to target clinically relevant mutations only, we performed a prediction of the variant’s effect on the protein function and included only those variants with potentially damaging effect. Furthermore, only variants with a high impact, e.g. missense or stop-gain variants, were retained, thereby removing variants with likely limited effect on the protein. Subsequently, we filtered by the cohort prevalence of affected genes and the type of drug-trials the DGIs had been derived from. Finally, selected DGIs not present in an expert curated database were manually curated (see Fig. 1 and “Methods” for a detailed description) and transcriptome based drug-response scores (DRS) were calculated, if available.

### Variant calling compared to previous TCGA analysis

Variant calling from the 412 patients` tumor genomes resulted in an unfiltered variant count (UVC) of 201,937 genomic variations. Quality metrics identified a high overlap with previous variant calling from the TCGA-BLCA cohort e.g. with Robertson et al.^6^ (see supplements). With a median agreement of 98.3%, the identified SNVs in particular were highly similar to the previously documented analyses, despite using a more conservative approach than Robertson et al.^6^. For identified CNVs the median agreement with previously reported CNVs was 84.6%.

### Variant and drug filtering

DGIdb is the largest cumulative database for DGIs. It works as a widely used open source tool to collect DGIs by text mining from public resources, such as publications and databases^7^, of which several are expert-curated. By querying DGIdb, we identified 34,039 SNVs and CNVs with existing references for non-curated DGIs (in total 16.9% of UVC).

**Figure 2:**
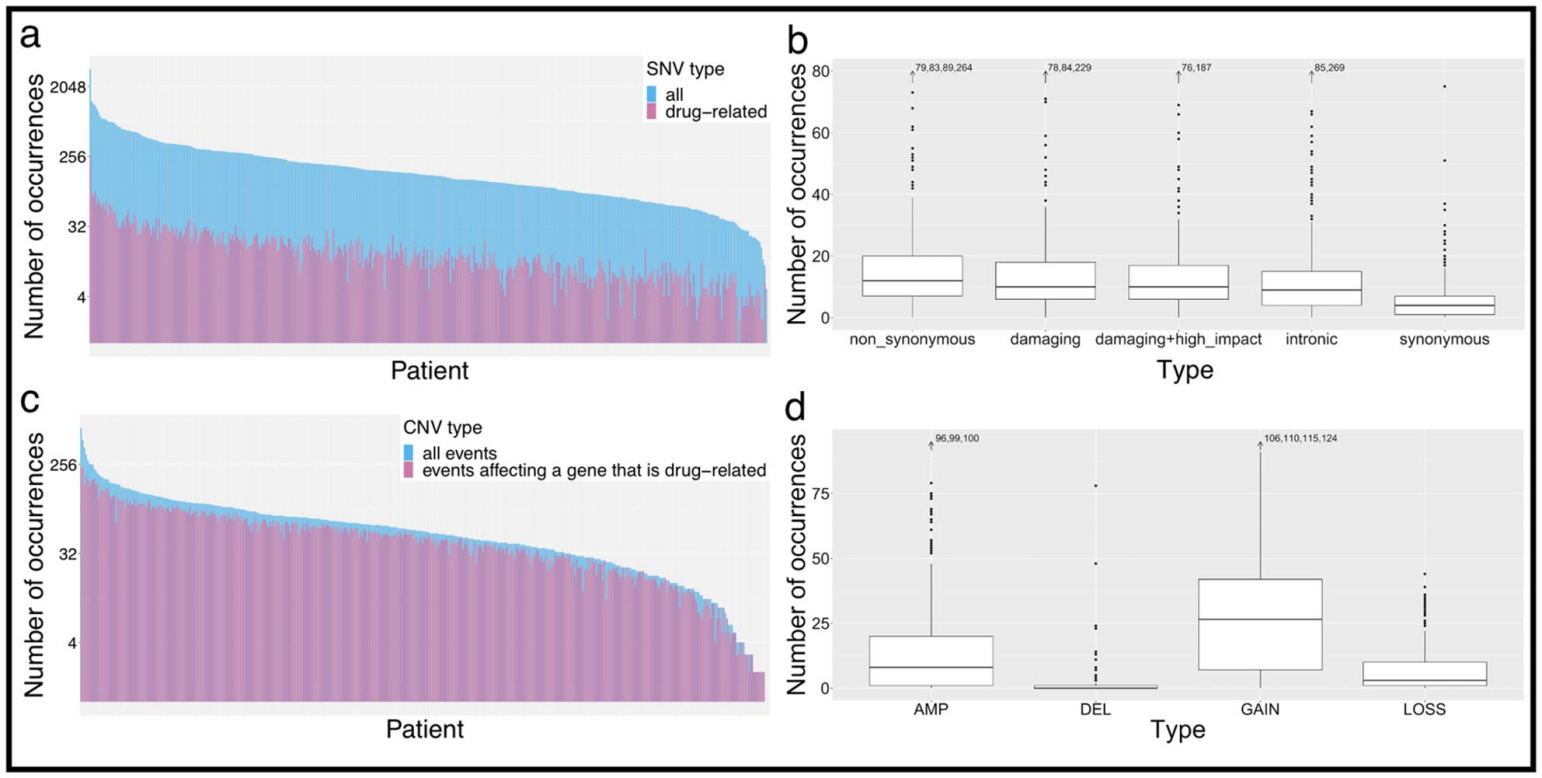
Filtering shows distinct effects on the SNV/CNV selection. (**a**) Barplot illustrates the log-scaled mutation load per sample for non-synonymous mutations, before (blue bars) and after (red bars) filtering for provided drug-gene interactions. Bars are overlying, not stacked. (**b**) Boxplots depict the distribution of SNV types after filtering for drug-related variants. (**c**) The barplot shows the differences of CNV load per sample throughout filtering. The overlying bars represent CNV load before (blue bars) and after (red bars) filtering for non-curated drug-gene interactions. (**d**) Boxplots illustrate the distribution of CNV types across TCGA-BLCA samples after filtering for drug-related CNVs (*DEL* deletion, 0 copies; *LOSS* copy number loss, 1 copy; *GAIN* copy number gain, 3–4 copies; *AMP* amplification, > 4 copies). (R Core Team (2019). R: A language and environment for statistical computing. R Foundation for Statistical Computing, Vienna, Austria. https://www.R-project.org/)^57^.

Before DGIdb-based filtering, tumor samples showed a wide range of mutational load from 1 to 7320 SNVs (median 293). The vast majority of samples presented drug-related SNVs after filtering, ranging from 0 to 574, with a median of 23 mutations (see Fig. 2a). Variants fulfilling the subsequent filtering steps to only select damaging SNVs with high predicted impact on the protein, still presented a large subset of non-synonymous SNVs (see Fig. 2b). The CNV count per sample changed from 0 to 595 (median 52) prior to filtering, to 0 to 243 (median 44) after (see Fig. 2c). Copy number gains and amplifications outweighed copy number losses and deep deletions (see Fig. 2d). Thus, here the applied filters to only select deep deletions or amplifications led to a minor decrease in CNVs evaluated for treatment prediction.

**Figure 3:**
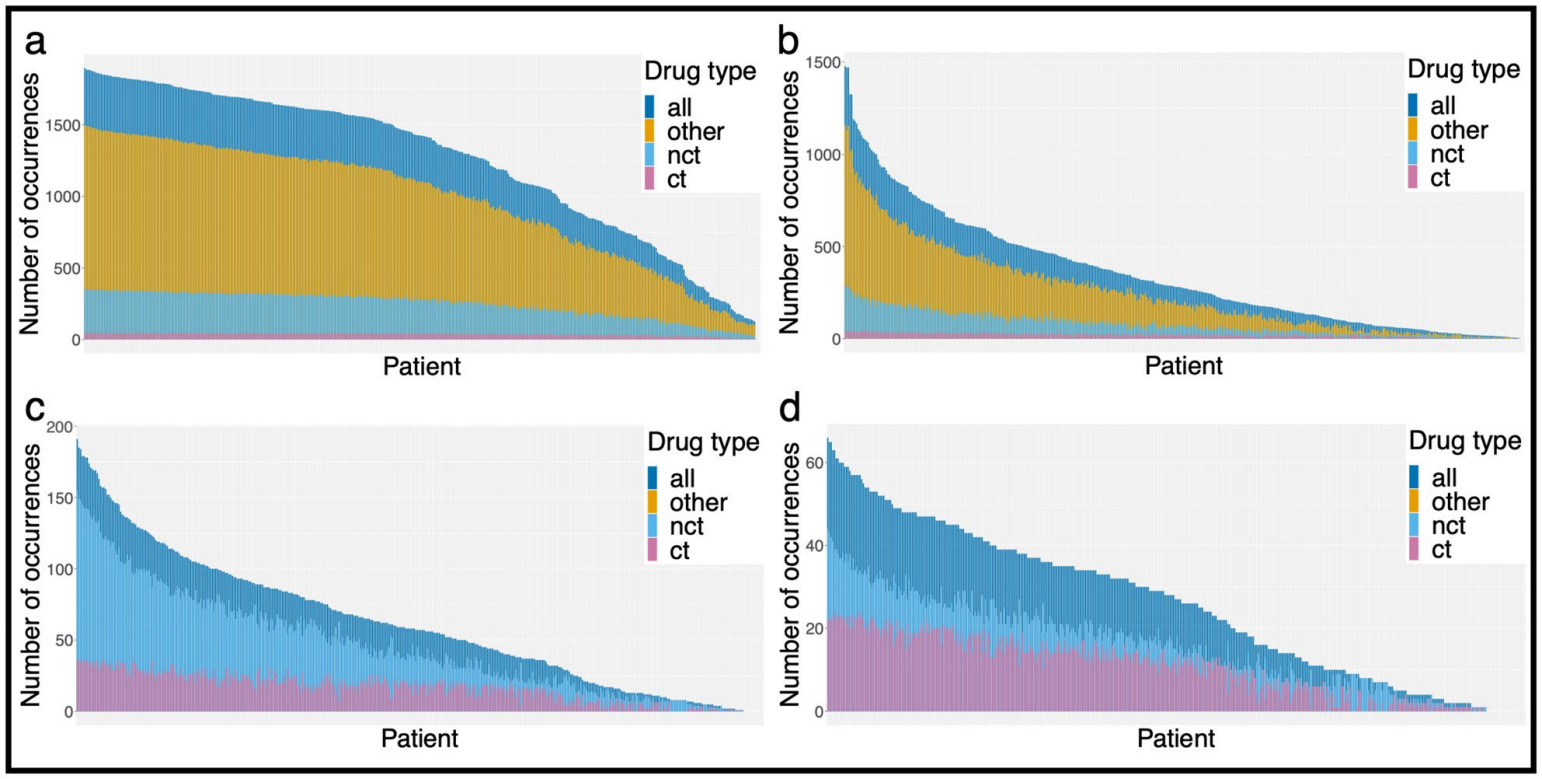
Development of the “drug load” along the filtering process. Each bar depicts a “drug load” per patient. Bars are not stacked, but overlying. Note that the y-axes change throughout the filtering. Drugs tested in bladder cancer trials or trials generally including solid tumors (ct) were distinguished from drugs used in general cancer trials (nct) and from medications not related to cancer in general. Purple bars illustrate the total drug load per sample. Drugs with no reference to cancer therapy are illustrated in yellow (other). (**a**) Barplot illustrates the “drug load” for all SNVs and CNVs after querying DGIdb before any filters were applied. In (**b**) “drug load” after filtering for clinically relevant variants is depicted. In (**c**) cohort prevalence filters for variants were applied and drugs with no reference to cancer therapy were excluded. (**d**) Barplot illustrates the final “drug load” for curated drug-gene interactions after integration of CIViC and manual curation results. (R Core Team (2019). R: A language and environment for statistical computing. R Foundation for Statistical Computing, Vienna, Austria. https://www.R-project.org/)^57^.

Next, we assigned the non-curated DGIs to the individual samples. This resulted in a “drug load” per sample, representing all predicted drugs with a gene-dependent recommendation. Figure 3 illustrates the effect of each step of filtering on the “drug load”. The combined “drug load” considering all drugs for all SNVs and CNVs varied from 0 to 1896 (median 1422) (Fig. 3a). Filtering DGIs for the selected variants with predicted clinical relevance (high transcriptome impact score) reduced the “drug load” to 0–1478 (median 274) drugs per sample (Fig. 3b). Applying cohort prevalence filters and excluding drugs without relation to cancer therapy resulted in 0–191 (median 57) drugs per sample (Fig. 3c). Manual curation and the CIViC (“Clinical Interpretation of Variants in Cancer”; https://civicdb.org) query created a final total “drug load” of 0–66 drugs (median 27) per sample (Fig. 3d).

**Figure 4:**
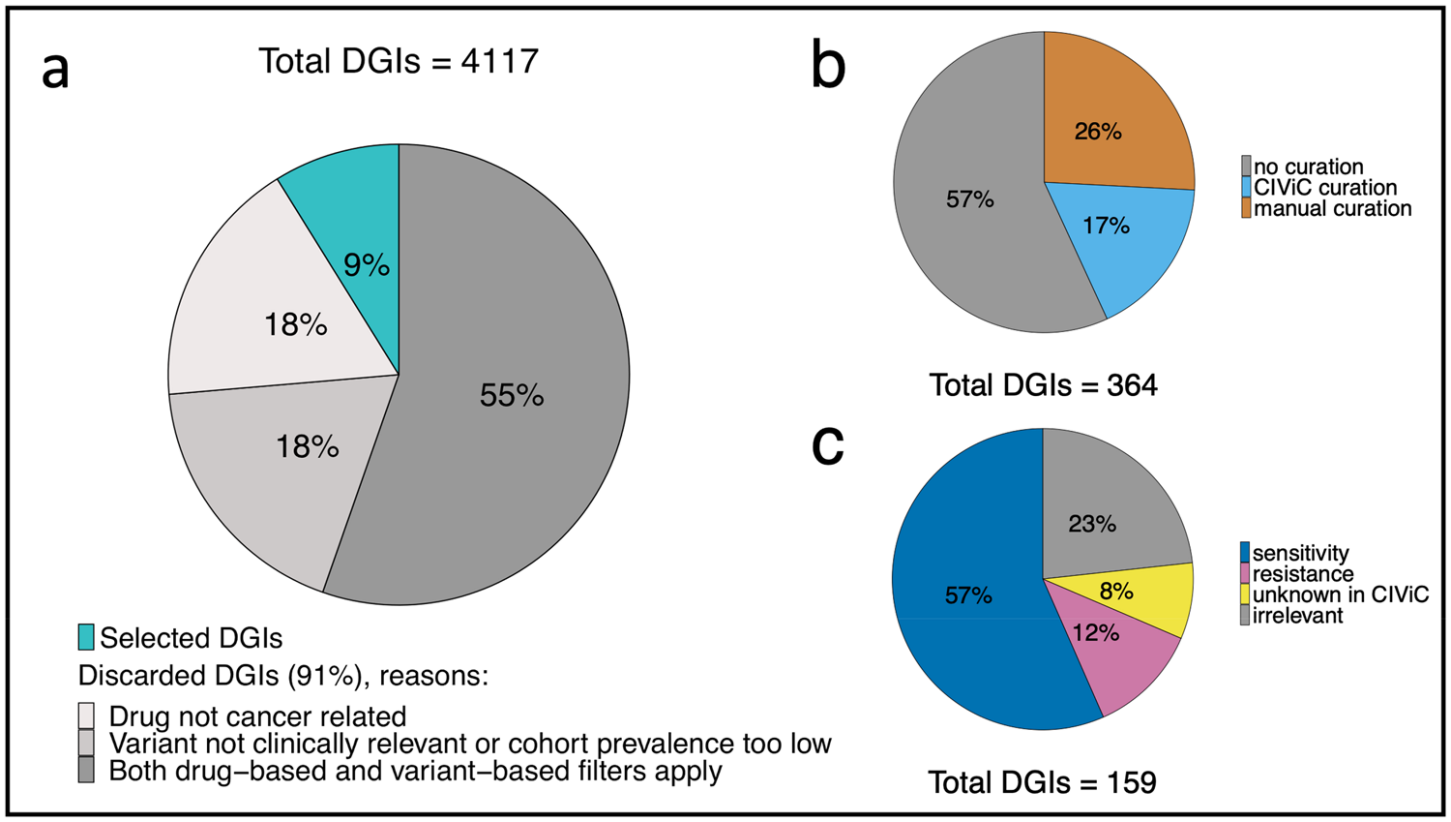
Influence of automated and manual filtering on the number of drug-gene interactions (DGIs). (**a**) The pie chart illustrates the fraction of DGIs filtered in an automated fashion, grouped by filter criteria. In (**b**), the fractions of DGIs that underwent curation or CIViC query are shown (note that not all available DGIs were manually curated, as this was beyond the scope of this study), whereas in (**c**), the assessment categories of the manually or CIViC curated DGIs are depicted. Note that combination treatments are excluded in this plot, since they all refer to the same DGIs and would thus be counted twice. (R Core Team (2019). R: A language and environment for statistical computing. R Foundation for Statistical Computing, Vienna, Austria. https://www.R-project.org/)^57^.

From the remaining 11,938 variants present across the cohort (6% of UVC), we demanded a variant cohort prevalence of 15%, which resulted in a set of 109 genes and 207 drugs (for details see suppl. Fig. S2). Thus, of the initial 4117 DGIs 91% were dismissed during automated filtering before manual curation and CIViC query. A majority of drop out resulted from both variant- and drug-related criteria. For instance, drugs not being related to cancer therapy caused the dismissal of 18% of DGIs. Low cohort prevalence and non-damaging variants resulted in the removal of a further 18% (see Figs. 3, 4a).

### Manual curation and CIViC integration

Up to this point in our investigation the workflow was fully automated (see Fig. 1a,b). The selected DGIs resulted in a set of 109 genes and 207 associated drugs (see suppl. Fig. S1). However, the level of evidence of selected DGIs could be separated into two groups. The first group was supported by CIViC DGI references. CIViC is an open source database for expert curated DGI references, which we integrated into our workflow to be automatically queried^8^. The second group was compiled of non-curated DGIdb entries and lacks information on the actual support of the treatment. To base our in silico predictions on proven DGIs only, we conducted a manual curation of non-curated DGIs (see “Manual curation of drug–gene interaction”). Note that we selected all SNV-dependent non-curated DGIs for manual curation. Manual curation is very time consuming and we expected similar methodological results for CNV-dependent DGIs. The non-curated SNV-dependent DGIs selected for manual curation represent 26% of all selected DGIs (see Fig. 4b). It can be expected that the fraction of non-curated DGIs will decrease over time, with more comprehensive information available in databases such as CIViC.

Interestingly, manual curation uncovered 62% of all investigated DGI references to be irrelevant, due to missing evidence of single agent efficacy or missing references to drug or gene itself. Commonalities of sources without proper reference to the DGI of interest include administration of the drug as part of a therapy regimen (which does not allow for evaluation of the single agent efficacy) and exclusive citations of either drug or gene in the list of references. In respect to all manually curated DGIs, 23% proved to be irrelevant due to exclusion in manual curation.

By extracting information concerning the direction of the DGI (sensitivity/resistance) from the DGI references, we were able to create binary predictions of response direction. While a majority of 57% of DGIs described sensitivity reactions, 12% of DGIs referred to resistance and 8% contained conflicting evidence (see Fig. 4c). Additionally, we used information from the mined references to annotate DGIs and discovered 10 reasonable combination therapies. Finally, filtering out DGIs neither supported by CIViC information nor by manual curation, reduced the number to 21 final genes and 72 drug/drug combinations.

To exemplify the results of the manual curation we depict examples for each possible categorie of annotation (SENSITIVITY/RESISTANCE/UNKNOWN).

A SENSITIVITY for veliparib is predicted by our algorithm, amongst others, in case of an ATM mutation due to the work of Subhash et al.^9^. In their study on gastric cancer they identified sensitivity for veliparib in case of ATM loss-of-function mutations of ATM^9^.

Yonesake et al. identified a mechanism of resistance to cetuximab in case of ERBB2 activating mutations^10^. Their study was called by our algorithm as a non-curated reference, manual curation led to the annotation of RESISTANCE in case of mutation^10^.

The efficacy of a DGI cannot be evaluated, if, within the referred study, the drug was administered within a therapy regime but not as a single acting agent, e.g. for a DGIdb reference for an interaction between PIK3CA and fluorouracil^11^. An non-curated DGI for FGFR3 and ponatinib was called on the results of the study “Combined targeting of FGFR2 and mTOR by ponatinib and ridaforolimus results in synergistic antitumor activity in FGFR2 mutant endometrial cancer models” by Gozgit et al.^12^. The call was a result of the DGIdb query algorithm, which found FGFR3 mentioned in the running text of the study but without any reference to an interaction to ponatinib^12^. In the aforementioned cases DGIs were annotated as UNKNOWN and dropped out from further utilization and analysis.

We want to point out that due to the fluid variables of our algorithm (entries in DGIdb, CIViC) in the future the dataset is likely to consist of partially different references.

### Integration of transcriptomic data

**Figure 5:**
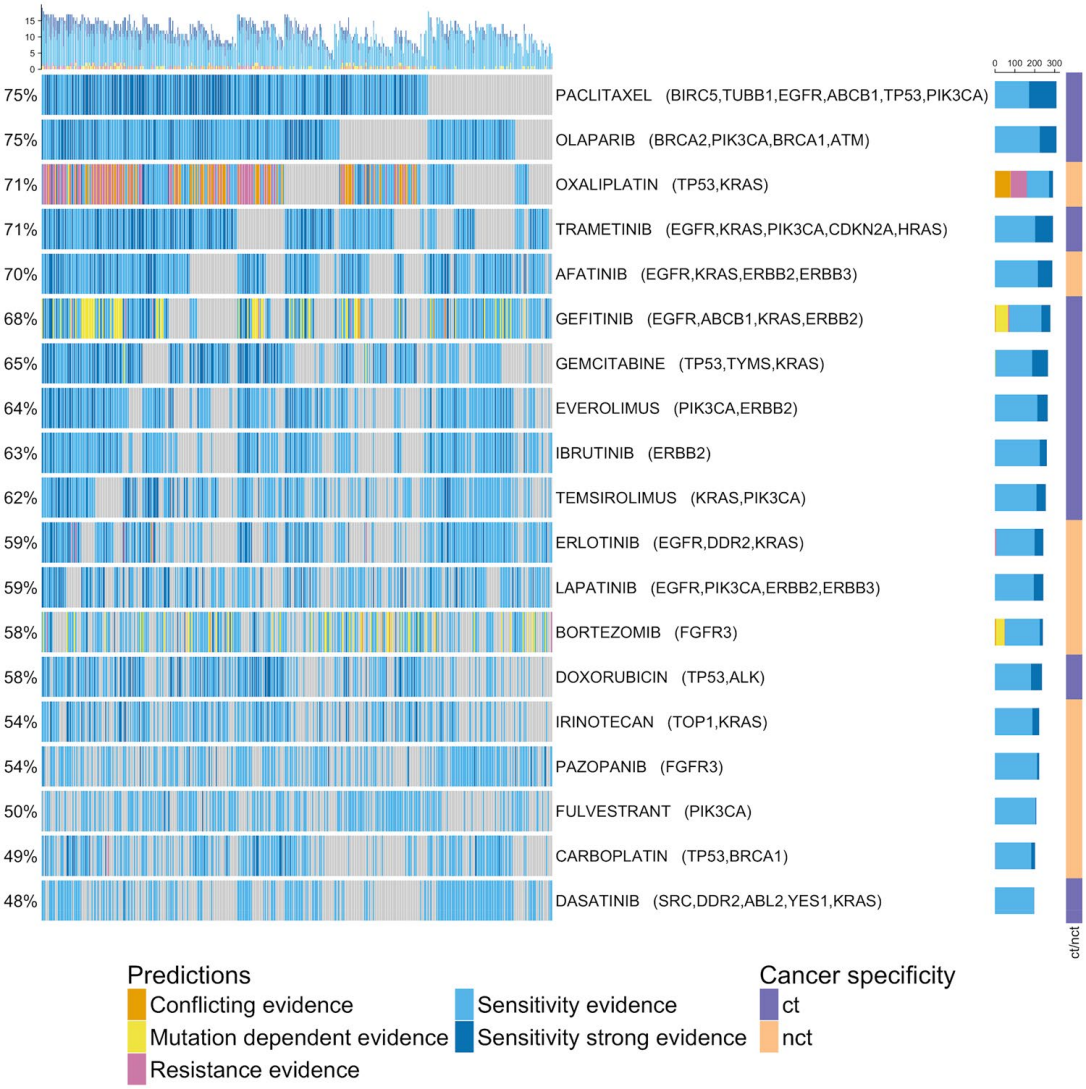
Oncoprint shows weighted evidence across samples based on drug response score (DRS) and curation support. Interacting genes are listed behind each drug. The direction/evidence categories are composed as following: Conflicting evidence: resistance prediction + DRS positive; Mutation dependent evidence: gene-dependent prediction + DRS positive/gene-dependent prediction + no DRS; Resistance evidence: resistance prediction + no DRS; Sensitivity evidence: sensitivity unspecific prediction + no DRS/sensitivity prediction + no DRS/no genomic + DRS positive; Sensitivity strong evidence: sensitivity unspecific prediction + DRS positive/sensitivity prediction + DRS positive. “ct” and “nct” refer to cancer-type specific and non-cancer-type specific as defined above. (R Core Team (2019). R: A language and environment for statistical computing. R Foundation for Statistical Computing, Vienna, Austria. https://www.R-project.org/)^57^.

To further consolidate the genomic-level predictions, we used transcriptome-dependent drug response scores (DRS) derived from the NCI-60 cell lines. The DRS were applied on each individual sample. DRS were measured for 26% (19/72) of the finally selected drugs (see Fig. 5 and suppl. Fig. S2). Note that a positive DRS indicates that cell-line samples responded favorably to the tested drug depending on their level of gene expression, i.e. a positive DRS is interpreted as sensitivity. However, it is important to note that a negative DRS does not implicitly mean that the cell line sample was resistant to the applied drug, but rather that no sensitivity could be observed (also see “Integration of DRS”).

### Final drug and gene panel

**Figure 6:**
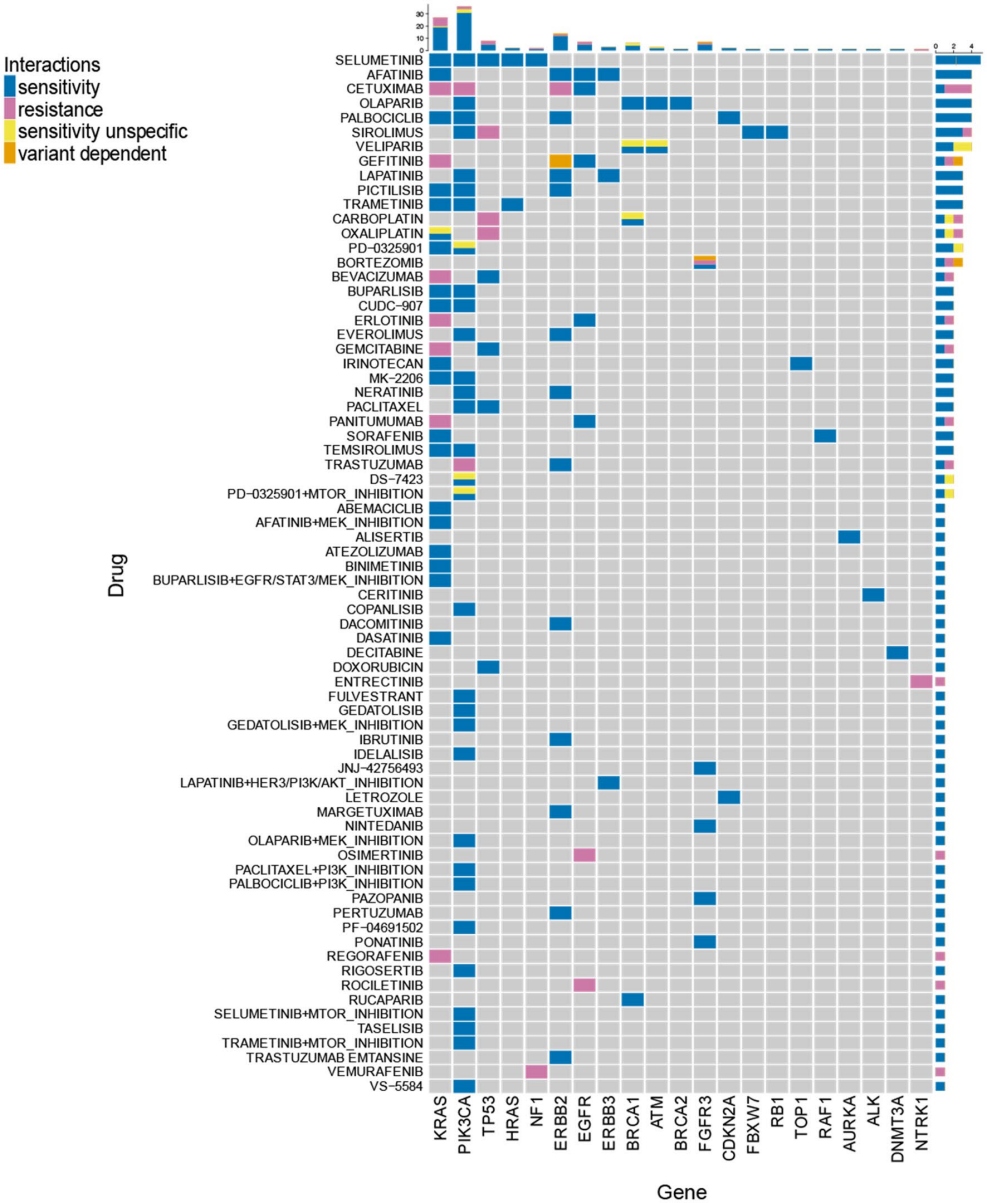
Our drug response prediction workflow discovers a panel of genes and related drugs/drug-combinations for personalized therapy in bladder cancer. The figure illustrates the final selection of drug-gene interactions (including combination treatments). “Sensitivity” and “resistance” refer to predicted sensitivity and resistance when the associated gene contains a respective variant. “Sensitivity unspecific” refers to genes where sensitivity to a particular drug could not be assigned to a particular variant but multiple variants would lead to the prediction. In “variant dependent” predictions, different variants within one gene can cause a divergent direction of the response prediction (sensitivity/resistance). (R Core Team (2019). R: A language and environment for statistical computing. R Foundation for Statistical Computing, Vienna, Austria. https://www.R-project.org/)^57^.

Based on the previously described steps, we identified a set of 21 actionable genes and 72 corresponding drugs or drug combinations as promising target-driven therapy suggestions for muscle invasive bladder cancer (see Fig. 6; suppl. Figs. S3, S4). In total 95% of TCGA-BLCA samples could be covered by our set of selected drugs and genes by being represented with at least one predicted DGI.

The finally selected set represents a mix of genes and drugs known in the context of bladder cancer, together with interesting potential new targets. 45 of the identified 72 drugs or drug combinations are currently or formerly tested in BLCA clinical trials. While being tested in solid tumor basket trials or other solid tumor entities, at the time of our investigation 17 of the listed drugs have not been tested in explicit BLCA trials yet (ceritinib; CUDC-907 (fimepinostat); dacomitinib; DS-7423; entrectinib; gedatolisib; idelalisib; letrozole; margetuximab; MK-2206; PD-0325901 (mirdametinib); PF-04691502; pictilisib; ponatinib; rigosertib; rociletinib; VS-5584)^13^. Twelve predicted drugs are being investigated in the MATCH trial^14^, five in the My Pathway trial^15^ and 12 in the COXEN trial^16^. As expected, only a minority of 12.5% (9/72) of drug approaches are non-targeted therapies.

For ten DGIs reasonable combinational therapies could be identified to avoid therapy evasion. Furthermore, at the time of investigation we found six of the identified combinational therapy approaches in clinical trials with mostly solid tumors (buparlisib + MEK-inhibition; gedatolisib + MEK-inhibition; lapatinib + AKT-inhibition; paclitaxel + PI3K-inhibition; palbociclib + PI3K/mTOR-inhibition; selumetinib + mTOR-inhibition; trametinib + mTOR-inhibition)^13^.

The majority of the identified genes (16/21) are currently subject of clinical trials as prognostic markers or in reference to therapeutic decision making for BLCA therapy. However, at the time of our investigation DNMT3A, FBXW7, HRAS, RAF1 and TOP1 are not yet found as genomic markers in BLCA related clinical trials and we are the first to report them as potential candidates for marker-driven therapy testing^13^. With FGFR3 we identified the first and as yet only genomic marker with a dependent FDA-approved drug for locally advanced and metastatic BLCA^17^ amongst our predictions.

### Divergence in drug response prediction

Within a majority of samples, drug response predictions depended on multiple different mutations. In each individual sample a directed response prediction (sensitivity/resistance) for one drug could depend on different genes and therefore divergence of the direction of prediction was possible (in the following referred to as “gene-dependent divergence”). In total, 252 pairs of drug and sample contained any kind of divergence, referring to 158 individual samples with multiple divergent predictions. We note that in a majority of samples 55% (228/412) the drug response predictions did not show any divergence. The 252 gene-dependent divergences found across the samples can be categorized into 122 within CIViC predictions, 97 within manual curation, and 33 resulting from differences between CIViC and manual curation predictions (2 in CIViC: resistance/manual curation: support; 31 in CIViC: support/manual curation: resistance). Thus, in all of these cases multiple genes had been mutated that had different impacts on the drug response. We identified divergent predictions exclusively resulting in reference to 8 drugs (docetaxel; sirolimus; cetuximab; bevacizumab; lapatinib; oxaliplatin; gemcitabine; trastuzumab). Between-source divergences corresponded to 3 drugs (cetuximab; sirolimus; gemcitabine). In total, predictions in only 1.2% (5/412) of samples included between-source divergence (arising exclusively from the curation of different DGIs across the curation sources).

### Molecular subtypes and drug response prediction

We investigated whether the 21 genes from the identified gene panel as well as the 89 drugs with DRS information show different prevalence in the previously described molecular subtypes of bladder cancer from the consensus work from Kamoun et al.^18^. Among the identified genes, logistic regression and ANOVA analysis detected 9 genes with significant mutation prevalence differences across subtypes (see suppl. Figs. S6, S7), based on a significance level of p value < 0.01 after multiple testing correction. Of 89 drugs with available DRS information, 83 drugs showed significant prediction differences between molecular subtypes (see suppl. Fig. S8a,b), based on a Kruskal–Wallis rank sum test with a significance level of p value < 0.01 after multiple testing correction.

### Potential application of the drug and gene panel

The drug and gene panel resulting from our workflow is a groundwork for a personalized therapy testing approach for BLCA. Investigating the predicted drugs, we see agents that have been clinically tested in BLCA, such as EGFR-inhibitors like cetuximab. Furthermore, mTOR-inhibitors like sirolimus, agents affecting the RAF/MEK/ERK pathway such as selumetinib, or PARP-inhibitors such as olaparib appear in our panel. With 10 identified drug candidates, PI3K-inhibitors form a major group of which only three (buparlisib; copanlisib; taselisib) are currently in clinical testing explicitly in BLCA.

**Figure 7:**
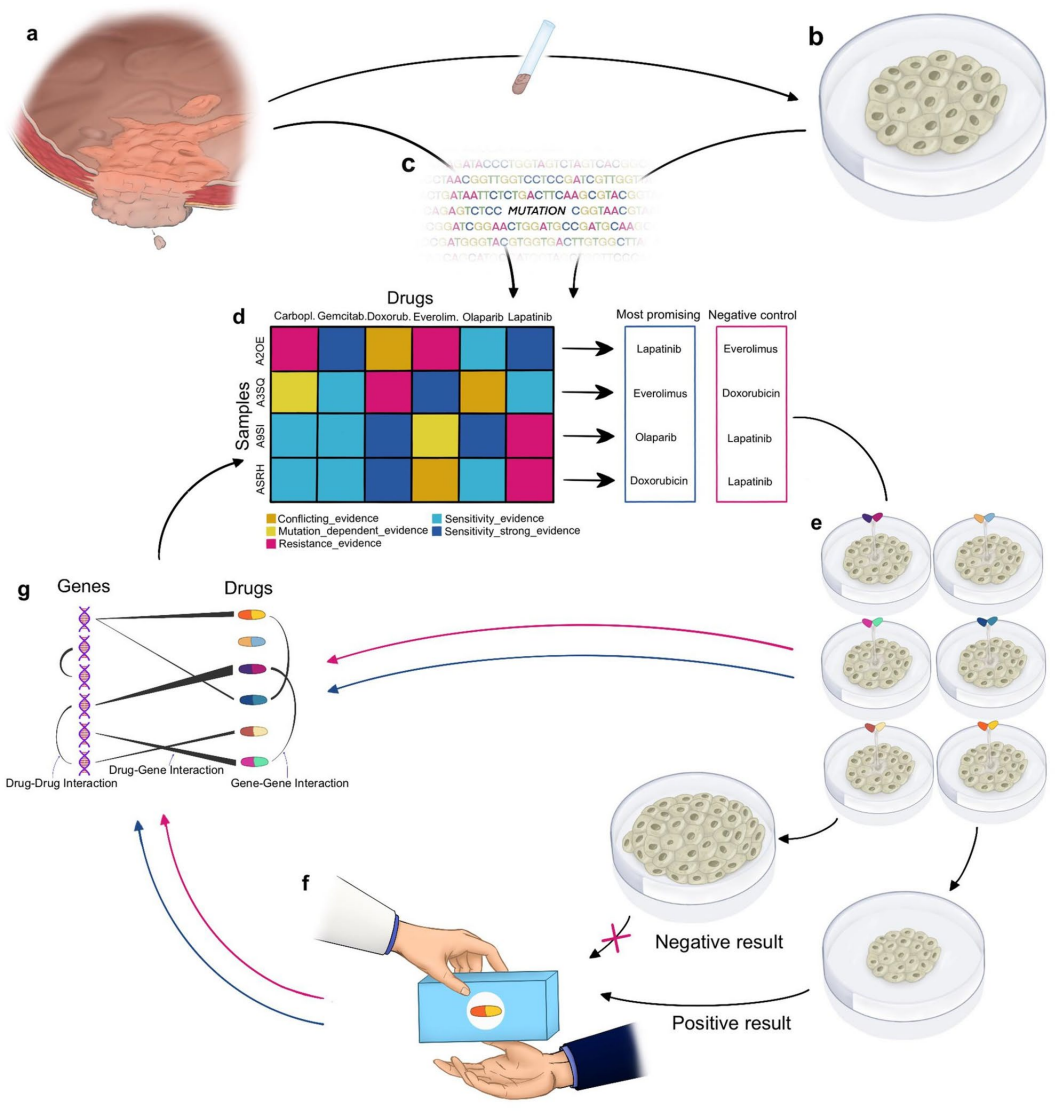
Blueprint for future clinical applications. (**a**) Histologic samples are derived from the tumor mass and processed into (**b**) individual patient derived tumor models. (**c**) Tumor mass and tumor models undergo sequencing to confirm genomic comparability. (**d**) Sequencing results are analyzed with our algorithm for drug response predictions. Delivering weighted (level of support) and directed (sensitivity/resistance) predictions, the most promising sensitivities can be selected to be tested against predicted resistances, functioning as negative controls. (**e**) The derived proposals are tested in the patient derived models. (**f**) The drug with the best response is administered to the patient, while feedback about in vitro sensitivities and resistances is reported to the database. (**g**) Clinical response is also reported to databases (DGIdb; CIViC etc.), increasing or decreasing the power of the DGIs for the next query of the algorithm. (Procreate, Version X, https://procreate.art).

Figure 7 illustrates a blueprint for the future application of our workflow. The gene and drug panel can serve as a cost-efficient yet comprehensive molecular diagnostics that is tailored to BLCA tumors. To illustrate an example, in the case of a significant PI3K-mutation (found in 38.8% (160/412) of all samples; see suppl Fig. S3) our algorithm predicted variant-dependent sensitivity to 22 drugs and 6 drug combinations (see Fig. 6). Additionally, three unspecific gene-dependent predictions for sensitivities were found alongside two predicted resistances. Both resistances refer to EGFR-inhibitors; cetuximab and trastuzumab. Moreover, predictions from our algorithm can be weighted in reference to the level of supporting evidence (only genomic/genomic + DRS information available) (compare Fig. 3; suppl. Fig. S2). Subsequently, testing of the predicted drug response in patient derived tumor models can generate several implications for future clinical administration (see Fig. 7e). The efficacy of predicted sensitivities from drugs of the same group (e.g. the PI3K-inhibitors buparlisib and gedatolisib) can be assessed preclinically. Similarly, the observed resistance predictions can be utilized as negative controls in testing the aforementioned tumor model. Both in vitro and in vivo results would mandatorily be reported to the database, the algorithm was interrogating at the outset, to increase or decrease the power of the underlying DGIs.

## Discussion

Poor and unforeseeable outcomes of standard therapies in BLCA raise the question, whether empiric “one size fits all” therapy and testing approaches can be justified in the setting of notably individual tumors^7^. Indeed, the genomic and transcriptomic diversity in BLCA offers a plethora of known actionable targets^6^. For other cancer types single genomic aberrations have proven to be relevant therapy targets, e.g. BRAF mutations in melanoma or HER2 mutations in breast cancer^19,20^. In contrast to single marker-driven diagnostics and decisions, molecular tumor boards in the US (Mayo Clinic^21^; Weill Cornell^22^) as well as in Europe (DKFZ/NCT^23^; ETH Zurich/SwissMTB^24^) started to comprehensively investigate individual molecular tumor landscapes. They examined clinical knowledge across oncological entities resulting in (off-label) drug suggestions for these often end-of-treatment-line patients, with notable responses^24^. Despite known targetable mutations, except of recently approved FGFR-inhibitors^17^ further genomic marker-driven therapies have yet to prove efficacy in BLCA. Therefore, BLCA investigations in comprehensive marker-based drug response predictions are highly warranted.

This study introduces an algorithm to facilitate a personalized and highly automated genome- and transcriptome-dependent in silico drug response prediction in BLCA. Furthermore, it identifies and avoids pitfalls of database derived drug response predictions in personalized medicine. Our workflow identified 21 genes corresponding to 72 drugs and drug combinations as candidates for genomic marker-driven therapy in BLCA. The majority of the identified drugs (45/72) are currently the subject of research for targeted therapy in BLCA and other cancer types. Importantly, even without any a priori focus, our algorithm identified the single approved genomic marker for targeted therapy in bladder cancer, FGFR3^13^. This may reinforce that our workflow is a valid approach for the identification of potentially relevant DGIs. Since the method was similarly applied to all genes, we can highlight the discovery of DNMT3A, FBXW7, HRAS, RAF1 and TOP1 as potential candidates for mutation-dependent pharmacotherapy, not having been reported in clinical studies up to this point^13^.

Alongside the identification of new potential targets, our WES-based approach could increase sensitivity towards existing targets. Prior analyses using panel-based sequencing methods identified up to 60% predefined targetable lesions in BLCA patients^25^. Our WES-based approach identified one or more of such lesions in 95% of muscle-invasive BLCA in the TCGA cohort. Furthermore, transcriptomic data added another level of evidence to our predictions. Additional evidence on the transcriptome level is an even stronger indicator that (1) the variant observed on the genomic level has an impact and (2) that the drug actually has the predicted effect. Therefore, our algorithm offers a robust strategy for the prediction of promising targets in most patients with muscle-invasive BLCA.

Within the group of identified targetable alterations, we found examples of altered genes playing notable parts in the pathogenesis of BLCA. DNA methylation regulation is crucially dependent on the DNMT3A gene product. Its mutations are described in many cancer entities^22^. In BLCA, mutated DNMT3A is known to cause hypermethylation and thereby silence promoters of tumor suppressor genes^23^. So far it is not reported as a genomic marker for a targeted therapy approach in BLCA. In the case of DNMT3A activating mutations, our algorithm acknowledged this pathogenetic mechanism and suggested use of the DNA methyltransferase inhibitor decitabine.

The potential importance of combinational therapies in personalized drug discovery became obvious during manual curation. In case of a PIK3CA mutation, our algorithm suggested CDK4/CDK6-inhibitor palbociclib. CDK4/CDK6 is affected downstream in the line of the PI3K (= PIK3CA) pathway, mediated by AKT and Cyclin-D. Due to according references with synergistic efficacy of a combined inhibition of PI3K and CDK4/CDK6, e.g. in breast cancer models, manual curation suggested PI3K-inhibition in combination with palbociclib in case of PIK3CA mutation^26^.

Drug filtering and manual curation after the identification of DGIs using DGIdb.org uncovered several key findings. We have found that a majority of DGIs were dismissed due to both drug and gene related reasons. Drugs were dismissed due to an absence of evidence as cancer therapies; they were for example investigated in drug metabolism instead. We suggest that databases for disease-drug-gene relations, instead of drug-gene interactions only, would address this problem. Filtering thresholds caused some DGIs to be dismissed due to low cohort prevalence. These thresholds were chosen to create a drug and gene panel of manageable size. However, these excluded DGIs could be of future interest. Ongoing research and the ever-increasing evidence base of expert curated databases such as CIViC will eventually overcome cohort prevalence barriers.

During manual curation many non-curated DGIs had to be dismissed due to missing evidence supporting the provided DGI. For instance, simple occurence of the drug or gene name in the list of references of a quoted article led to a non-curated DGI. Such DGIs were dismissed because said article might not have studied the efficacy of this DGI. Automation and introduction of artificial intelligence may solve the problem of vague DGIs. In 2019 Kim et al. introduced the artificial intelligence based search engine for disease-gene-chemical relationships DigChem, which is able to avoid aforementioned text-mining errors. However, their study emphasized that the overlap with other DGI databases (CTD^27^, IBM Watson for Drug Discovery^28^ and DrugBank^29^) is complementary, rather than displaying directly comparable results^30^. Moreover, in the process of DGI filtering we found a large disparity between provided DGIs and the ratio of expert curated DGIs. Therefore, we conclude that manual curation has still to be considered mandatory to achieve evidence-based DGIs.

Although only in a minority of samples, we identified divergent drug response predictions due to the presence of multiple DGIs per sample and due to non-matching genome- and transcriptome-based predictions. A majority of divergences resulted from drugs with multiple-gene dependent response predictions within a given sample. In these cases model and clinical testing will facilitate judgment about the dominant DGI. Importantly, at the time of investigation a majority of samples were free of divergences in drug response prediction.

Both curations, manual and CIViC based, identified more sensitivities than resistances in DGIs. Although validation is missing so far, we credit this fact to a known reporting bias for positive study results e.g. sensitivities^31^. For the creation of comprehensive DGI databases a more liberal reporting of resistances will be essential. Siu et al. have addressed the need for liberal and barrier-free reporting of DGIs in creating a scheme for responsible sharing of cancer genome data for utilization in comprehensive analysis^32^. Therefore, testing and reporting of drug resistance in DGIs as consequently as existing reporting of drug sensitivities, will be of paramount importance. Integration of proven and disproven drug-gene interactions into open source databases will be of utmost importance to improve drug response predictions. Prospectively, our workflow will include these growing databases and consequently predict promising drugs contemporaneously.

A subtype specific investigation of the prevalence of the referred genes identified significant differences for 9 genes. While this might hint towards an explanation for a different clinical behaviour of the molecular subtypes to standard therapies^5^, the low prevalence of the genes within the subtypes (see suppl. Figs. S6, S7) underlines the subtype heterogeneity. Subsequently, alongside supporting the predictive value of molecular subtypes for clinical decision making, our results underline the necessity for an even finer granularity of therapy selection criteria for a truly personalized therapy.

Biologically, advanced tumor stages are characterized by the consequences of multiple prior therapies and of progressed intratumor heterogeneity. Taking tumor evolution theories into account a plethora of potential therapy evasion mechanisms might have developed by that time and create limitations to personalized therapy so far^33^. However, our algorithm can be adapted for testing in patient derived tumor models and thereby offers a tool to facilitate testing in multiple tumor stages and of multiple locations (e.g. metastases). The remarkable findings of Biswas et al.^34^, demonstrated mutational similarities between different evolutionary tumor branches. These findings might open the door to solutions how to address intratumor heterogeneity with targeted therapies. In our opinion, developing a dynamic understanding of oncogenesis and its underlying processes of therapy evasion development is compelling. It will allow for multi-time and multi-location evaluation of tumors and lead to accordingly individualized therapies.

We acknowledge that our approach is still hypothesis-generating and further validation is needed. Depending on databases which become extended by time, the results of our algorithm are fluent. We point out that in the future, querying our algorithm would deliver other results than by the time of our investigation. By evolving evidence our results can be supported, so that weak predictions turn into strong predictions and vice versa; novel predictions are possible caused by new results which are fed into the databases. At the same time, contradicting new evidence can enfeeble DGIs as well.

Hence, we want to underline the showcase character of this study. The fluent character of the algorithm is illustrated in Fig. 7d–g. Therefore, our algorithm can deliver an up-to-date base for mutation dependent personalized drug testing, rather than offering a more or less static truth. Ultimately, the in silico drug response predictions require further testing in patient derived models and clinical trials prior to implementation as personalized approaches in clinical practice. Importantly, clinical trials need to be designed in a genomic-marker aware fashion. In 2019, preliminary data from the BISCAY trial compared the efficacy of the PD-L1 checkpoint inhibitor durvalumab in combination with targeted therapies in selected urothelial cancer patients. Despite achieving a superior overall response rate with the combination of durvalumab and the PARP inhibitor olaparib in case of an DNA-damage-repair gene alteration, compared to durvalumab monotherapy none of the study arms could reach the threshold for a positive study result^35^. In 2017 Kiss et al. demonstrated that genomic variations and their impact as prognostic markers for therapy response, have to be carefully interpreted with consideration of transcriptional and gene-regulatory factors as well as of co-existing mutations^36^. These findings highlight the importance of marker-driven trials. Basket-trials such as the My Pathways study (NCT02091141) or the NCI-MATCH study (NCT02465060) offer access to individualized treatment with a diversity of targeted agents. Our algorithm represents a sophisticated tool that can be implemented in clinical trials and lay the foundation to precise cancer therapy based on a comprehensive understanding of molecular oncogenesis.

In conclusion, our algorithm for in silico drug response prediction in BLCA identified drugs with proven antitumor activity but importantly also new potentially druggable targets and novel drugs, which have not yet been tested in BLCA. Moreover, our drug prediction provided evidence for 95% of BLCA samples, highlighting its applicability to the majority of bladder cancer patients. Integration of genomic and transcriptomic data acknowledged different regulatory processes in oncogenesis and increased the granularity of our predictions. Importantly, our algorithm yielded an efficient and affordable gene panel for drug response prediction based on a comprehensive approach. While we showcased the potential in this in-depth study of the bladder cancer patient cohort of TCGA, the workflow itself is applicable to any cancer type. Other available TCGA cohorts will provide a very valuable resource to perform the proposed semi-automated drug response prediction that could indicate future therapies for a genomic-driven personalized cancer therapy.

## Methods

### The Cancer Genome Atlas bladder cancer cohort

We analyzed the genomic landscape of the 412 bladder cancer patients of the TCGA-BLCA cohort^6^. Based on the whole exome sequencing (WES) information, variants in the tumor genome have been identified and linked to their potentially targeting drugs. We prioritized variants and drugs according to their clinical relevance and prevalence in bladder cancer patients. The workflow is depicted in Fig. 1 and detailed in the following. The 412 patients in the TCGA-BLCA cohort present a well-mixed population, e.g. in regard to gender, BLCA subtype, and tumor stage, which will likely reduce the effect of clinical features on the drug prediction results.

For each patient in the TCGA-BLCA cohort, the WES-based bam files for normal as well as tumor samples have been downloaded from the GDC data portal^37^. Bam files had already been generated with the GDC data harmonization workflow^38^. For patients with multiple tumor and/or normal samples, we selected the sample with the later plate number according to the GDC guidelines^39^, and preferred fresh frozen over FFPE samples. Further, to ensure a sample selection as homogeneous as possible, we selected the blood derived normal whenever possible, as this was the normal control available for the majority of patients.

### Variant calling from the TCGA cohort

To investigate the genomic landscape of the 412 bladder cancer patients, we performed somatic variant calling for the identification of single nucleotide variants (SNVs), as well as copy number calling to identify copy number variants (CNVs). The pipeline for WES analysis from the retrieved bam files to unannotated variant calls is based on the framework described in Singer et al.^40^, employing the Snakemake workflow environment^41^.

#### SNVs

In brief, variants were called based on the GATK best practices workflow^42^, with somatic variant calling on the matched tumor and normal samples. To achieve high quality calls and reduce the number of false positives, we applied three different variant callers and only considered variants that were identified by at least two callers. The utilized variant callers were MuTect^43^, Strelka^44^, and VarScan2^45^, where the latter two not only perform single nucleotide variant calling, but also identify small insertions and deletions in the sample.

Variants were annotated using SNPeff^46^ and SNPsift^47^ and enriched with information on their presence in ClinVar^48^, COSMIC^49^, and dbSNP^50^, as well as their overall mutation impact (e.g. missense variant or synonymous variant). We also performed a functional annotation to assess the potential of each variant to be damaging for the protein function.

#### CNVs

We used the CNV caller Facets^51^ to call copy number variants on the matched tumor and normal samples for each patient. All CNVs were annotated to identify the affected genes.

### In silico drug prediction and selection

Similarly as described in Singer et al.^24^, we queried DGIdb^7,52,53^ to identify the drug-gene interactions reported in a collection of 30 databases, of which several are expert-curated (e.g. MyCancerGenome^54^). The more databases support an interaction, the higher the scoring of the drug. Finally, based on drug-gene interactions identified in DGIdb we collected associated clinical trials at ClinicalTrials.gov. Here, we distinguished between clinical trials in BLCA and those also including solid tumors (cancer type, “ct”), non-cancer type specific (“nct”) trials, and trials not related to cancer in general (“other”). Trials in the “nct” category focus on patients with other cancer types, thus for bladder cancer patients their corresponding drugs would potentially be regarded as off-label therapy.

Integrating clinical trial information provides a first prioritization of the drug-gene interactions (DGIs) resulting from the DGIdb query. The initial set of possible drugs was filtered to only contain drugs that have previously been tested in cancer-related clinical trials (categories “ct” and “nct”). Further, we prioritized drugs according to their frequency in the cohort, to obtain a set of drugs that is likely to contain at least one possible match for each BLCA patient.

### Variant prioritization and selection

The set of identified variants (both SNVs and CNVs) was filtered to prioritize variants that are likely to be of clinical relevance, i.e. that (1) are likely to have an impact on the protein function and (2) that are associated with genes with DGIs listed in DGIdb. An overview of the filtering process is illustrated in Fig. 1. SNVs were filtered to only include those that have a high impact (higher than 20, based on the SNPeff impact list, see suppl. Table S1) and that are predicted to be damaging. The CNVs were filtered to only include those with deep deletions (copy number = 0) or high amplifications (copy number > 4).

Further, we only included variants that affect genes with a reported drug-gene interaction (based on the DGIdb query). Furthermore, we prioritized variants that occur more frequently across the TCGA-BLCA cohort, to obtain a final selection that is not only likely to be of clinical relevance, but also likely to have at least one targetable variant present in BLCA patients.

The minimum set cover analysis was performed to identify the fraction of the cohort that was covered by the selected set of priority genes (and drugs). It was based on in-house scripts and performed a greedy selection of the best possible set.

### Integration of CIVIC

The information provided by DGIdb is very comprehensive and thus a valuable resource to provide a basic set of DGIs as a starting point. However, it has several limitations that impede its direct use for clinically relevant in silico drug prediction. First of all, it is undirected and only lists DGIs without indicating the nature of the DGI, i.e. whether a mutation in the respective gene confers sensitivity or resistance. Second, it only reports interactions between genes and drugs and does not consider the individual variants present in a patient’s tumor. And finally, it contains not only expert-curated databases such as MyCancerGenome.org, but also comprehensive compound collections. As a consequence, we enriched the DGIdb based information with variant specific expert curated information from the CIViC database^8^. Here, individual aberrations that affect a specific gene are evaluated for their potential to confer sensitivity or resistance to a drug, and each evaluation (called evidence item) is rated with an evidence level to show the quality of the underlying information. For instance, preclinical studies are rated lower than information gained from multi-center clinical trials. Our CIViC based assessment of each DGI is accordingly applied on the variant level, such that in case different TCGA samples showed different variants of the same gene, a DGI can have divergent readouts, i.e. sensitivity or resistance. Note that it is possible that a variant-drug interaction type is not clearly identifiable from the information currently available in CIViC, e.g. in some studies a variant seemed to confer sensitivity while in other studies this was not a significant finding. In these “CONFLICT” cases we employ a majority vote that counts how many evidence items support sensitivity and resistance, respectively, and take the interaction type supported by the majority of evidence items. Note that before the majority vote is applied, we prioritize the evidence items according to their relevance, e.g. evidence items on bladder cancer would be prioritized over those with information from other cancer types. Further, CIViC contains evidence items that are not explicitly in favor of either sensitivity or resistance. Accordingly, the corresponding DGI is labeled as “UNKNOWN” to indicate that the DGI was found in CIViC, but that the available information is not sufficient to clearly predict response or resistance.

### Manual curation of drug–gene interaction

References of DGIs obtained from the DGIdb query with no annotation in the curated CIViC database were selected for manual curation. Clinical studies as well as experimental studies were included. First, categories for response were separated into “SENSITIVITY”, “RESISTANCE” and “UNKNOWN”. The category “UNKNOWN” thereby summarized all references with missing evidence of single agent activity, effectiveness or missing reference to the drug or gene itself. An annotation category was added to document restrictions such as: quality of data, conflicting evidence, variant dependent response, co-variant dependent response, gene expression dependent response, reasonable combinational therapy to avoid therapy evasion and clinically relevant information about the drug. Second, the full text articles referenced by DGIdb were screened for the gene and the active substance. If the active substance name was not found, generic names, brand names and accession numbers were searched for.

If gene and drug were found, data was screened to support single agent activity of the drug in clear reference to an explicit mutation and/or wild type. In clinical studies response “SENSITIVITY” was defined as improvement of response rate, progression of free survival or overall survival under administration of the drug, in case of mutated gene compared to wild type. Response “RESISTANCE” was here defined as decreased response rate, inferior progression free survival or overall survival under administration of the drug, in case of a mutated gene compared to wild type. In experimental studies, response “SENSITIVITY” was defined as deteriorated growth or decreased survival of the tumor model under administration of the drug, in case of mutated gene compared to wild type. Response “RESISTANCE” was defined as accelerated growth or increased survival of the tumor model under administration of the drug, in case of mutated gene compared to wild type. Response “UNKNOWN” was defined as no significant difference of clinical outcome or tumor model reaction when comparing mutant to wild type. References with missing evidence of single agent activity, effectiveness of the administered drug or missing reference to the drug or gene itself, were classified as response “UNKNOWN” as well.

### Integration of DRS

We used the CellMiner tool to mine for drug response correlating to expression data of the NCI-60 cell lines^55^. As previously published, corresponding genes were used to generate patient specific drug response scores (DRS) using correlation coefficients as weighting factors^56^. Thereby, DRS represent a statement on a potential drug response based on the level of expression of a certain gene product. Hence, a positive DRS is a prediction of a drug sensitivity, as the underlying investigations correlated tumor model response to a certain level of gene product expression. However, a negative DRS does not predict a therapy resistance, but a non-response to the drug.

### Molecular subtype analysis

To investigate potential differences in the distribution of the identified targetable genes across the molecular subtypes^18^, we performed logistic regression coupled to an ANOVA independently for each gene. We excluded samples not covered by the consensus definition (n = 6) as well as those assigned as “NE-like” (due to the small size of this group, n = 6). The null hypothesis for this test is that the molecular subtype has no influence on the mutation prevalence.

Using the Kruskal–Wallis rank sum test, we compared the DRS scores across the molecular subtypes independently for each available drug. For this analysis, we only considered the 405 patients having DRS information available and again excluded patients without a subtype definition and those belonging to the “NE-like” subtype, due to low sample sizes.

In both cases, multiple testing correction was applied using the Benjamini–Hochberg method.

## Supplementary Information

Supplementary information.

## Data Availability

The data that support the findings of this study are available from the corresponding author upon request.
