# Supplementary material for: A showcase study on personalized in silico drug response prediction based on the genetic landscape of muscle invasive bladder cancer
Source: Sci Rep. 2021 Mar 12;11:5849. doi: 10.1038/s41598-021-85151-3 (PMC7955125; doi:10.1038/s41598-021-85151-3)
Supplement: Supplementary file 1 — Supplementary information. [file 41598_2021_85151_MOESM1_ESM.docx]

A showcase study on personalized *in silico* drug response prediction based on the genetic landscape of muscle invasive bladder cancer

Friedemann Krentel ^1^, Franziska Singer ^2,3^, María Lourdes Rosano-Gonzalez ^2,3^, Ewan A. Gibb ^4^, Yang Liu ^4^, Elai Davicioni ^4^, Nicola Keller ^5^, Daniel J. Stekhoven ^2,3^, Marianna Kruithof-de Julio ^1^, Roland Seiler*^1^

^1^ Department of Urology, University of Bern, Switzerland

^2^ NEXUS Personalized Health Technologies, ETH Zurich, Switzerland

^3^ SIB Swiss Institute of Bioinformatics, Switzerland

^4^ GenomeDx Biosciences, Vancouver, Canada

^5^ University of Basel, Switzerland

Address correspondence to:

Roland Seiler, MD

Department of Urology

University of Bern

Bern 3010, Switzerland

[roland.seiler@insel.ch](mailto:roland.seiler@insel.ch) / [r_seiler@gmx.ch](mailto:r_seiler@gmx.ch)

**Keywords:** Muscle-invasive bladder cancer, Personalized therapy, Cancer diagnostics, Molecular diagnostics

# Supplements

## Quality metrics / overlap of variants with TCGA

As a basic comparison to the variant calling results published in the TCGA paper [^14^](https://www.zotero.org/google-docs/?u56D4b), we calculated the overlap between the variants identified with our workflow with the variants reported in the publication. Note that in the paper SNV information is provided for only 59 genes and CNV information is provided for only 33 genes. For CNVs, there are 2 sets of reported genes: 19 genes reporting only AMP/DEL events and 14 genes reporting only DEL/LOSS events.

For SNVs, to be comparable with the variants reported in the publication we used the set of variants remaining after application of the impact filter threshold. The overlap was calculated based on agreement between TCGA and our workflow on having a mutation vs having no mutation for the set of considered genes, across the 412 patients. On average, the agreement was 97.8%, with a median of 98.3% and an interquartile range (IQR) of 1.8%.

For CNVs, the overlap was calculated separately across both reported groups and to be comparable we mimicked the setting of the publication, e.g. only AMP/GAIN events were considered for the 19 “amp” genes while considering all remaining events as “not mutated”. A total of 4 patients were excluded from the comparison due to lack of available cnv data in TCGA publication. CNV data for genes E2F3 and SOX4 was reported jointly in the TCGA publication, which was accounted for when calculating the overlap (if any of the two genes matched the evaluated CNV type it was considered an overlap). Note that we filtered the CNV data to only include AMP and DEL events. On average, the publication and our workflow agreed in 83.7% of the cases, with a median of 84.6% and an IQR of 9.8%.


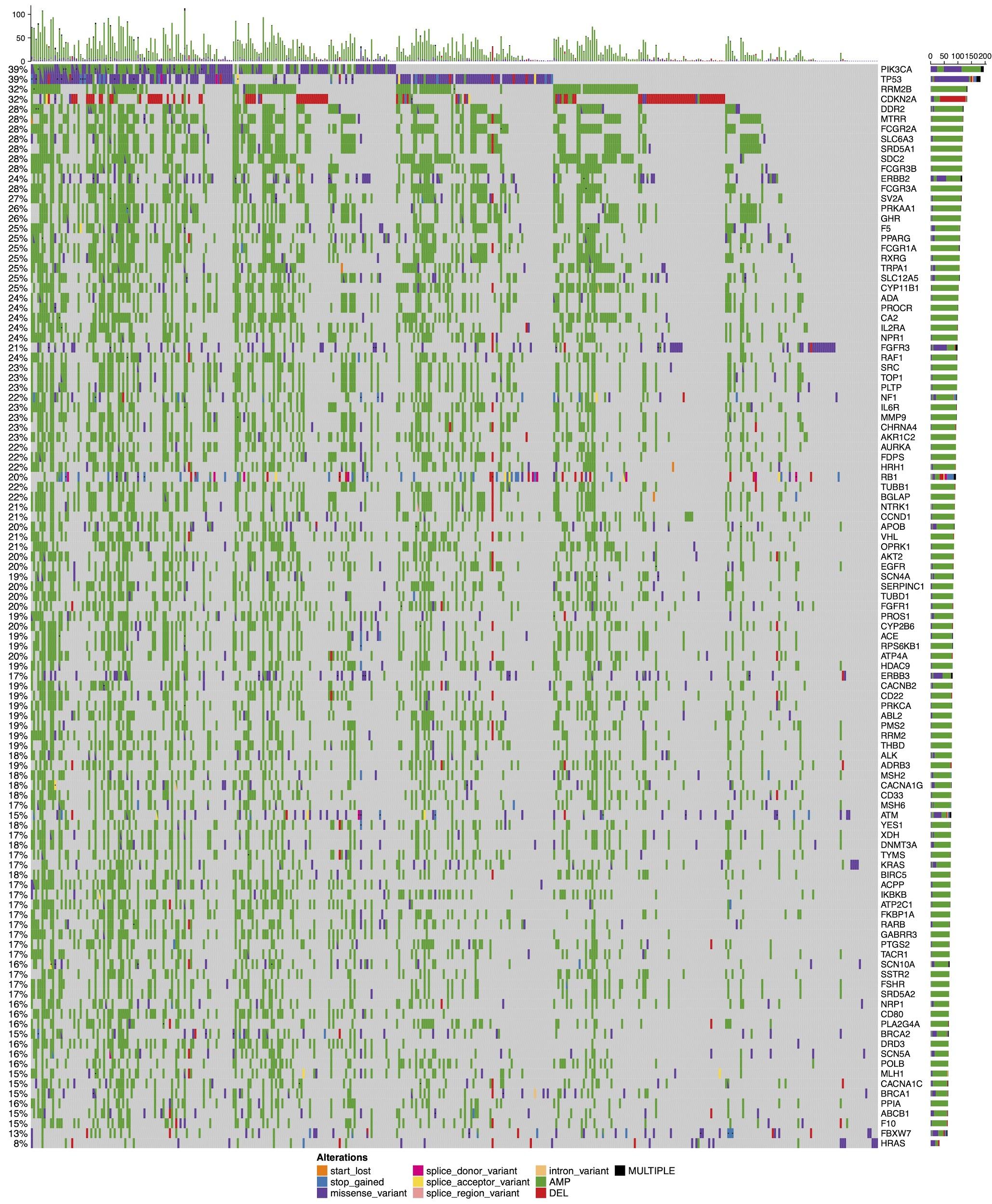


**Suppl. Fig. 1:** Oncoprint illustrates SNV and CNV prevalence within the 109 genes selected by fully automated filtering across the samples. (DEL = deletion; AMP = amplification, >4 copies). (R Core Team (2019). R: A language and environment for statistical computing. R Foundation for Statistical Computing, Vienna, Austria. URL: <https://www.R-project.org/>) ^57^

| **Annotation** | **Impact ordering** |
| --- | --- |
| structural_interaction_variant | 1 |
| protein_protein_contact | 2 |
| chromosome | 3 |
| gene_variant | 4 |
| non_coding_transcript_variant | 5 |
| non_coding_transcript_exon_variant | 6 |
| non_coding_exon_variant | 7 |
| intergenic_region | 8 |
| conserved_intergenic_variant | 9 |
| intragenic_variant | 10 |
| conserved_intron_variant | 11 |
| sequence_feature | 12 |
| custom | 13 |
| miRNA | 14 |
| regulatory_region_variant | 15 |
| TF_binding_site_variant | 16 |
| downstream_gene_variant | 17 |
| upstream_gene_variant | 18 |
| synonymous_variant | 19 |
| 5_prime_UTR_premature_start_codon_gain_variant | 20 |
| 3_prime_UTR_variant | 21 |
| 5_prime_UTR_variant | 22 |
| coding_sequence_variant | 23 |
| intron_variant | 24 |
| initiator_codon_variant+non_canonical_start_codon | 25 |
| initiator_codon_variant | 26 |
| stop_retained_variant | 27 |
| splice_region_variant | 28 |
| splice_branch_variant | 29 |
| 3_prime_UTR_truncation+exon_loss | 30 |
| 5_prime_UTR_truncation+exon_loss_variant | 31 |
| conservative_inframe_deletion | 32 |
| disruptive_inframe_deletion | 33 |
| conservative_inframe_insertion | 34 |
| disruptive_inframe_insertion | 35 |
| missense_variant | 36 |
| rare_amino_acid_variant | 37 |
| splice_donor_variant | 38 |
| splice_acceptor_variant | 39 |
| start_lost | 40 |
| stop_lost | 41 |
| stop_gained | 42 |
| frameshift_variant | 43 |
| exon_loss_variant | 44 |
| chromosome_number_variation | 45 |

**Suppl. Table 1:** SNPeff impact list. Ranking of variant impacts, where the “least deleterious” one is shown first and the “most deleterious” is shown last. Our prediction workflow applied a threshold of >20 for high-impact variant filtering.


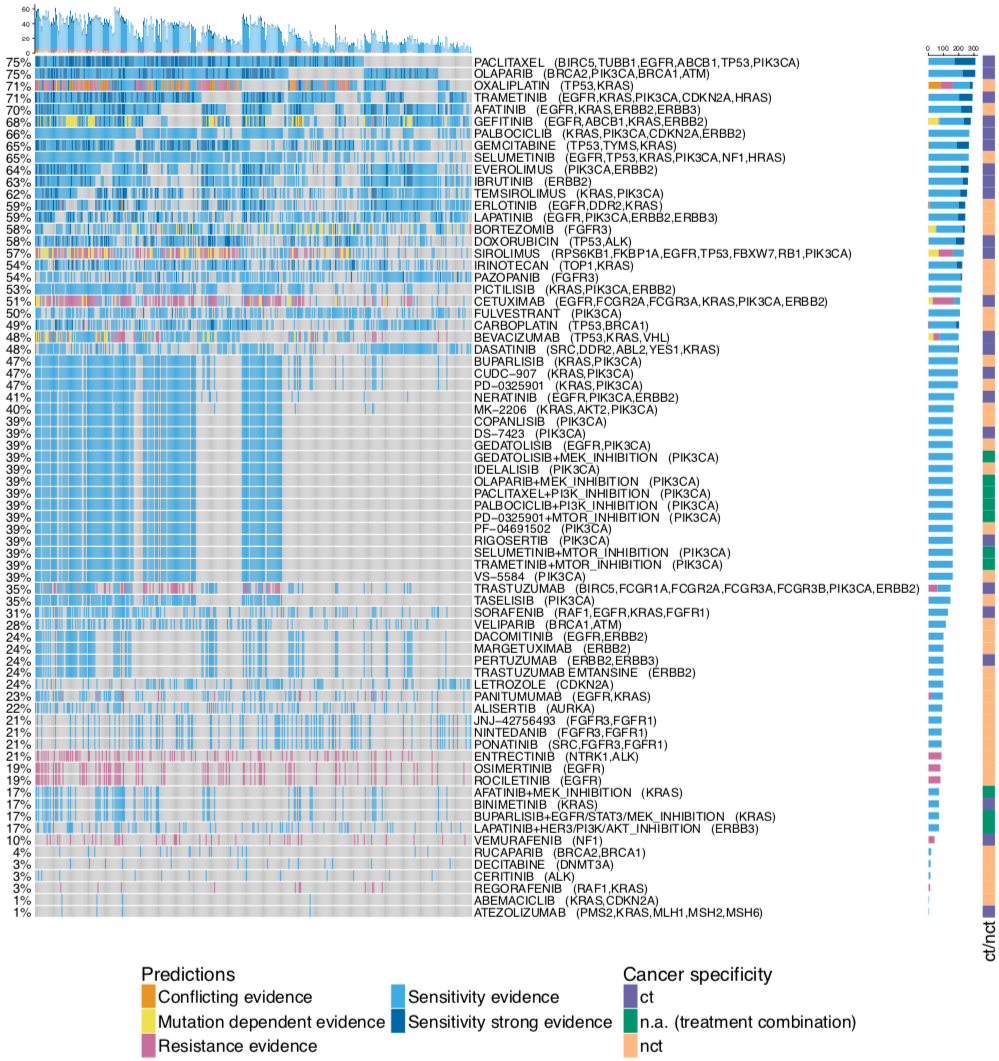


**Suppl. Fig. 2** Oncoprint shows weighted evidence across samples based on drug response score (DRS) and curation support for all drugs. Interacting genes are listed behind each drug. The direction/evidence categories are composed as following: Conflicting evidence: resistance prediction + DRS positive; Mutation dependent evidence: gene-dependent prediction + DRS positive / gene-dependent prediction + no DRS; Resistance evidence: resistance prediction + no DRS; Sensitivity evidence: sensitivity unspecific prediction + no DRS / sensitivity prediction + no DRS / no genomic + DRS positive; Sensitivity strong evidence: sensitivity unspecific prediction + DRS positive / sensitivity prediction + DRS positive. “ct” and “nct” refer to cancer-type specific and non-cancer-type specific as defined above. “n.a. (treatment combination)” refers to combination-therapies with according evidence derived from manual curation. (R Core Team (2019). R: A language and environment for statistical computing. R Foundation for Statistical Computing, Vienna, Austria. URL: <https://www.R-project.org/>) ^57^

**
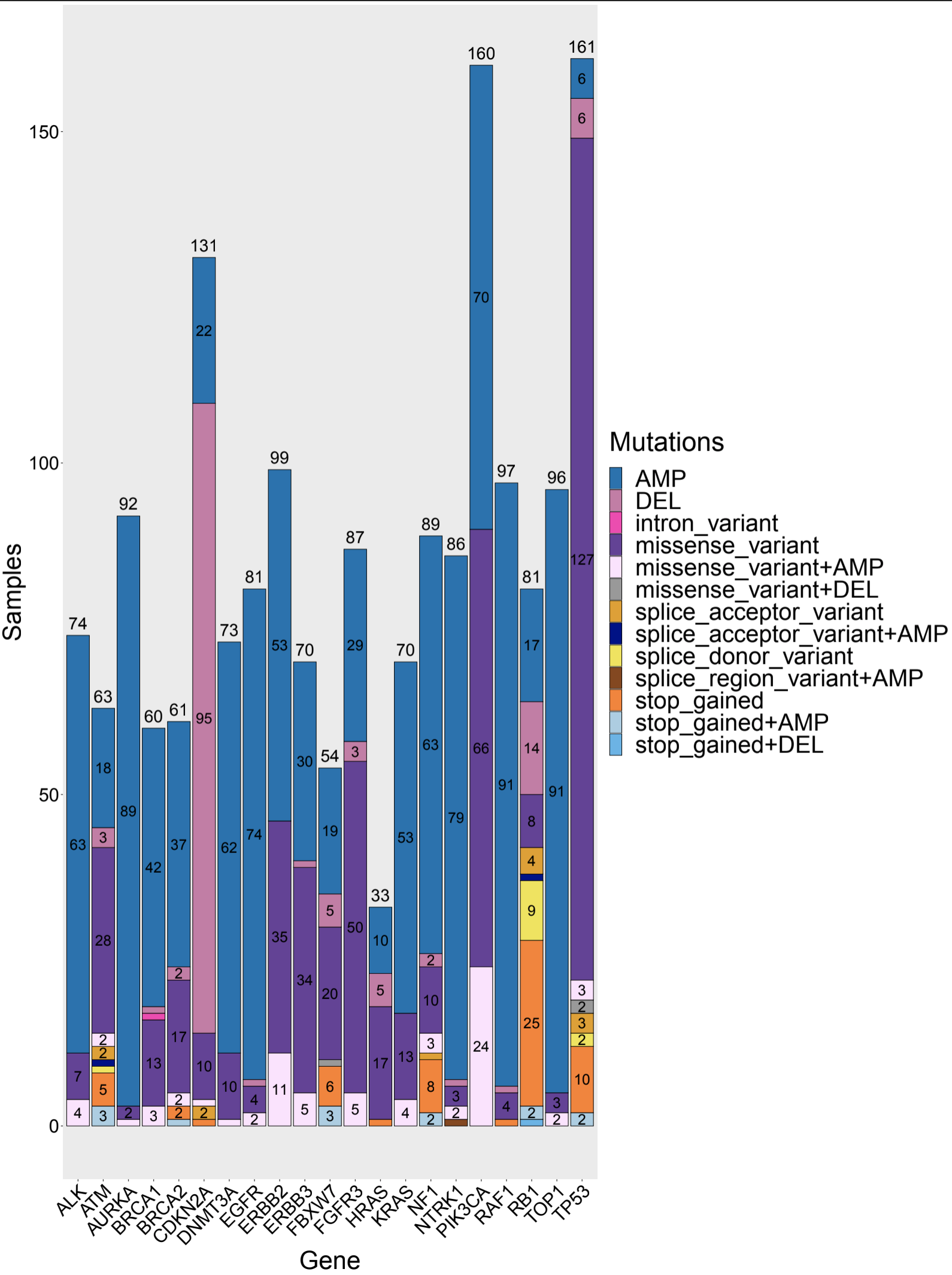
**

**Suppl. Fig. 3: Genes**

Information on observed variant types across the cohort for the final selection of 21 genes. Abbr.: AMP = Amplification; DEL = Deep deletion. (R Core Team (2019). R: A language and environment for statistical computing. R Foundation for Statistical Computing, Vienna, Austria. URL: <https://www.R-project.org/>) ^57^


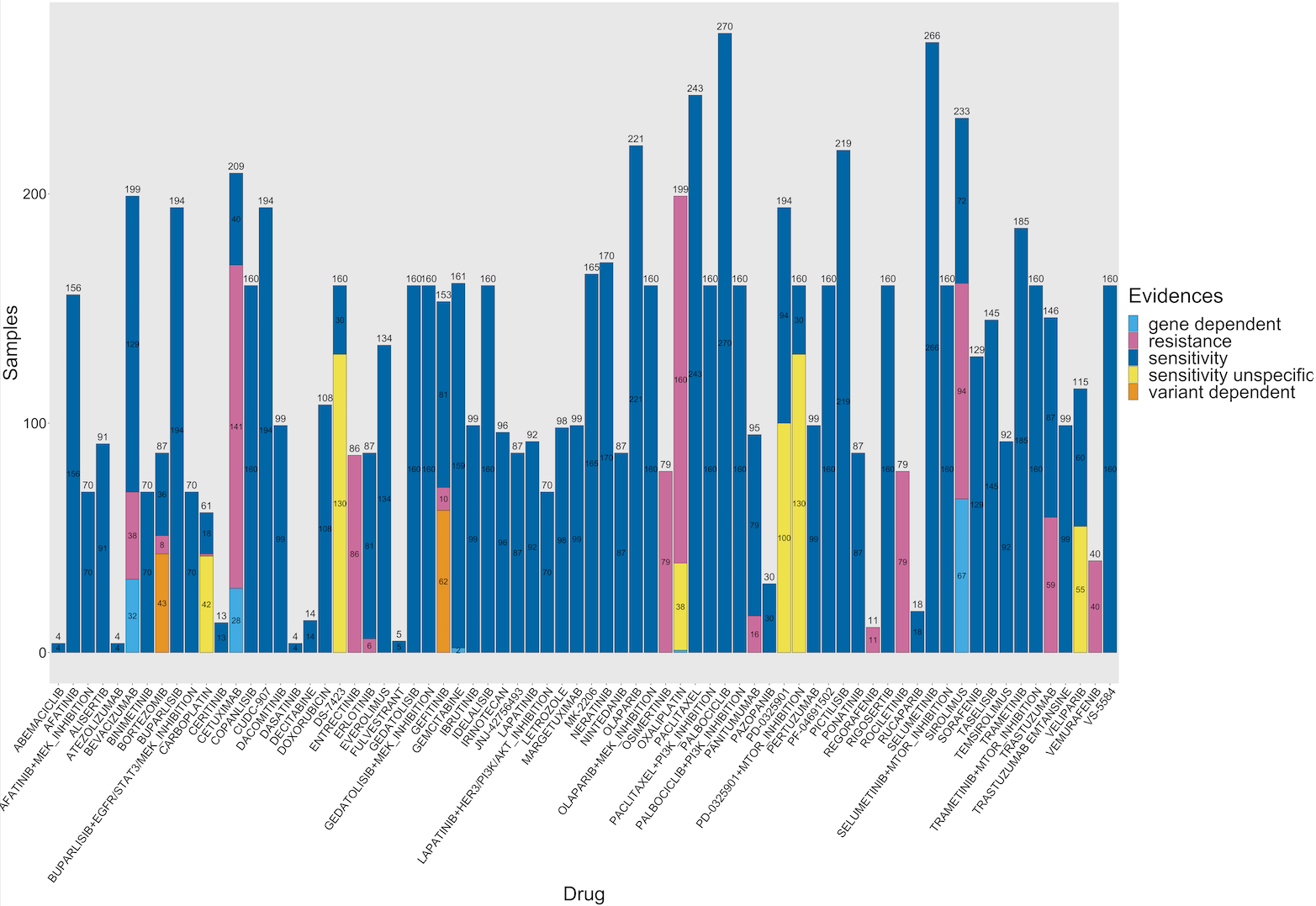


**Suppl. Fig. 4:** Curation information across all samples across all 72 drugs and drug combinations. Barplot showing the sample information for the final selection of 72 drugs. “Sensitivity” and “resistance” refer to predicted sensitivity and resistance when the associated gene contains a respective variant. “Sensitivity unspecific” refers to genes where sensitivity to a particular drug could not be assigned to a particular variant but multiple variants would lead to the prediction. “Gene dependent” categorizes samples that have multiple genes mutated that have a divergent (sensitivity/resistance) response to the same drug. In “variant dependent” predictions, different variants within one gene can cause a divergent direction of the response prediction (sensitivity/resistance). (R Core Team (2019). R: A language and environment for statistical computing. R Foundation for Statistical Computing, Vienna, Austria. URL: <https://www.R-project.org/>) ^57^


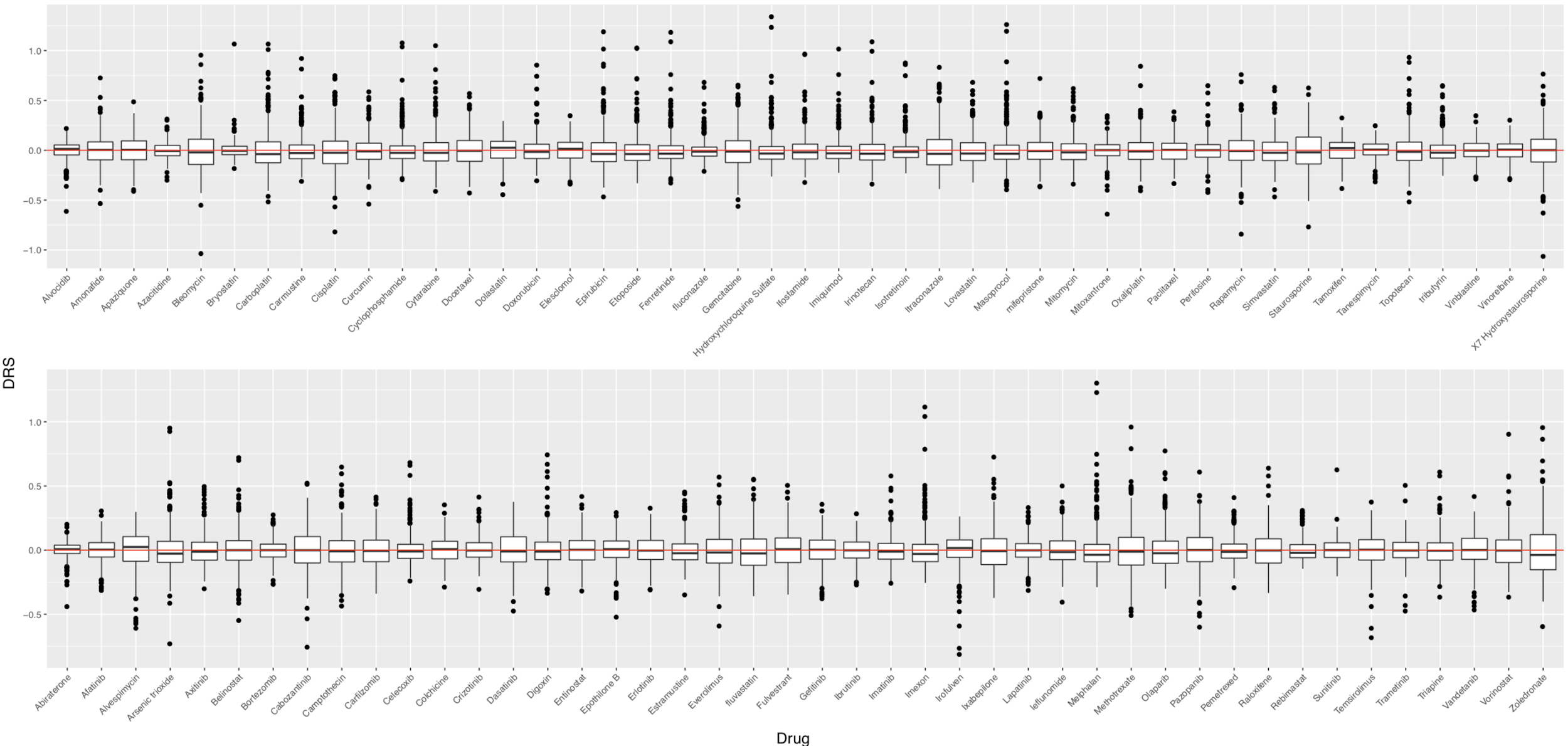


**Suppl. Fig. 5:** DRS score distribution per drug across the TCGA cohort (predictions available for 405/412 samples). (R Core Team (2019). R: A language and environment for statistical computing. R Foundation for Statistical Computing, Vienna, Austria. URL: <https://www.R-project.org/>) ^57^

**
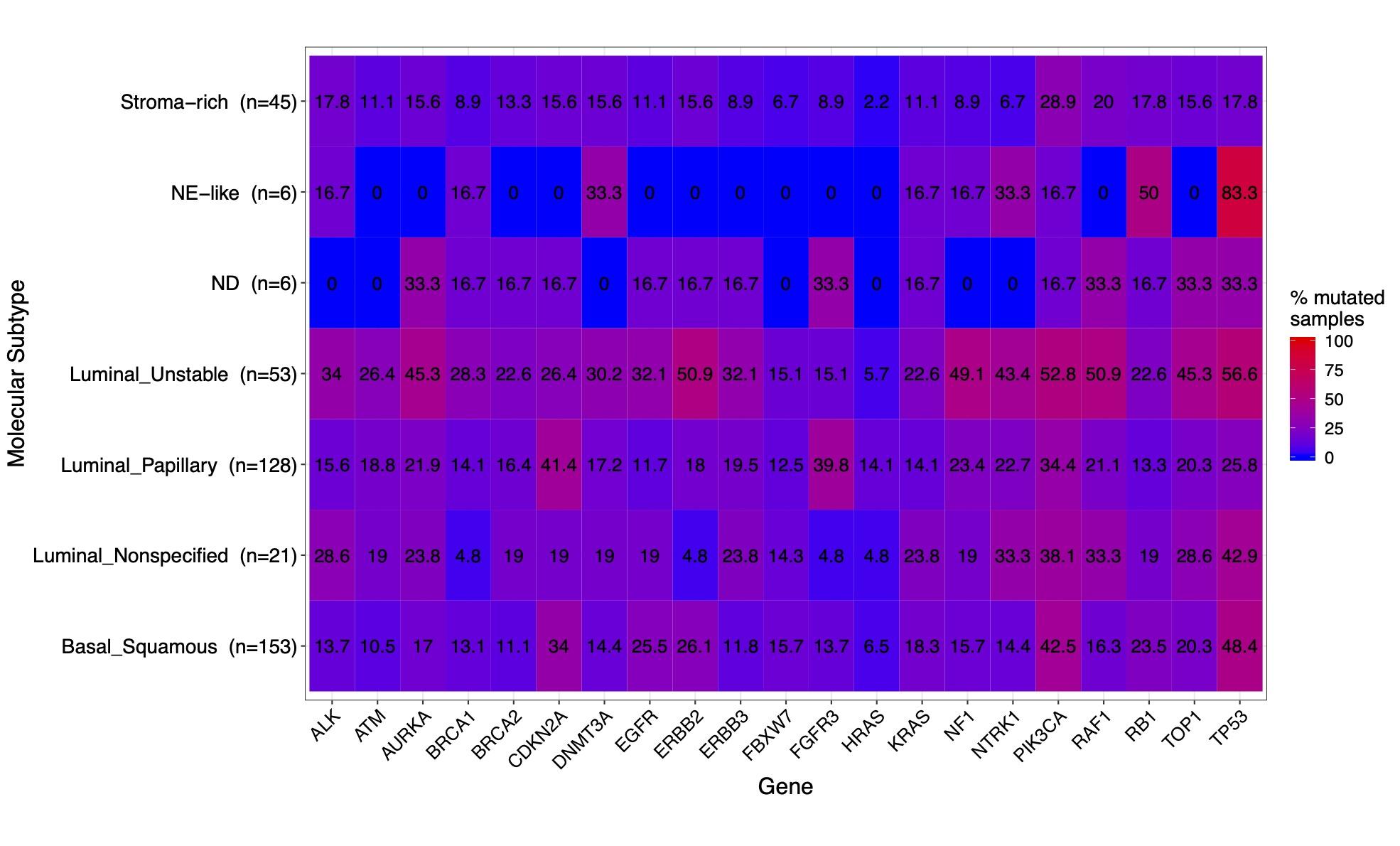
**

**Suppl. Fig. 6:** Distribution of the 21 identified genes across the six molecular subtypes from the 2020 consensus classification (Kamoun et al., European Urology, 2020). Note that “ND” corresponds to samples not covered by the consensus definition. (R Core Team (2019). R: A language and environment for statistical computing. R Foundation for Statistical Computing, Vienna, Austria. URL: <https://www.R-project.org/>) ^57^

**
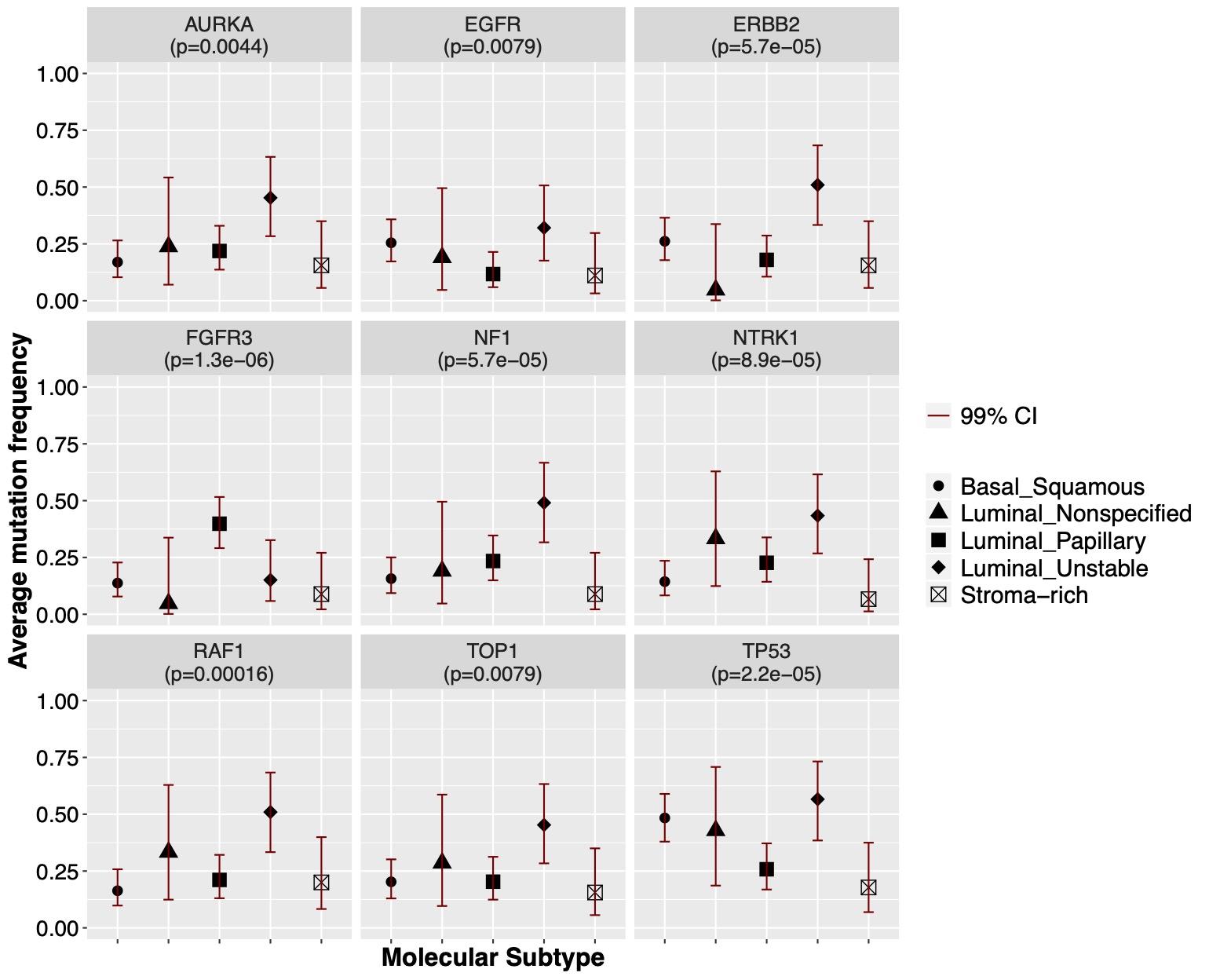
**

**Suppl. Fig. 7:** Logistic regression and subsequent ANOVA analysis identified significant prevalence differences for 9 of the identified genes in reference to molecular subtypes (Kamoun et al., European Urology, 2020). The scatter plots are presenting the mean proportion of mutated samples per subtype and the corresponding 99% confidence interval. (R Core Team (2019). R: A language and environment for statistical computing. R Foundation for Statistical Computing, Vienna, Austria. URL: <https://www.R-project.org/>) ^57^

**
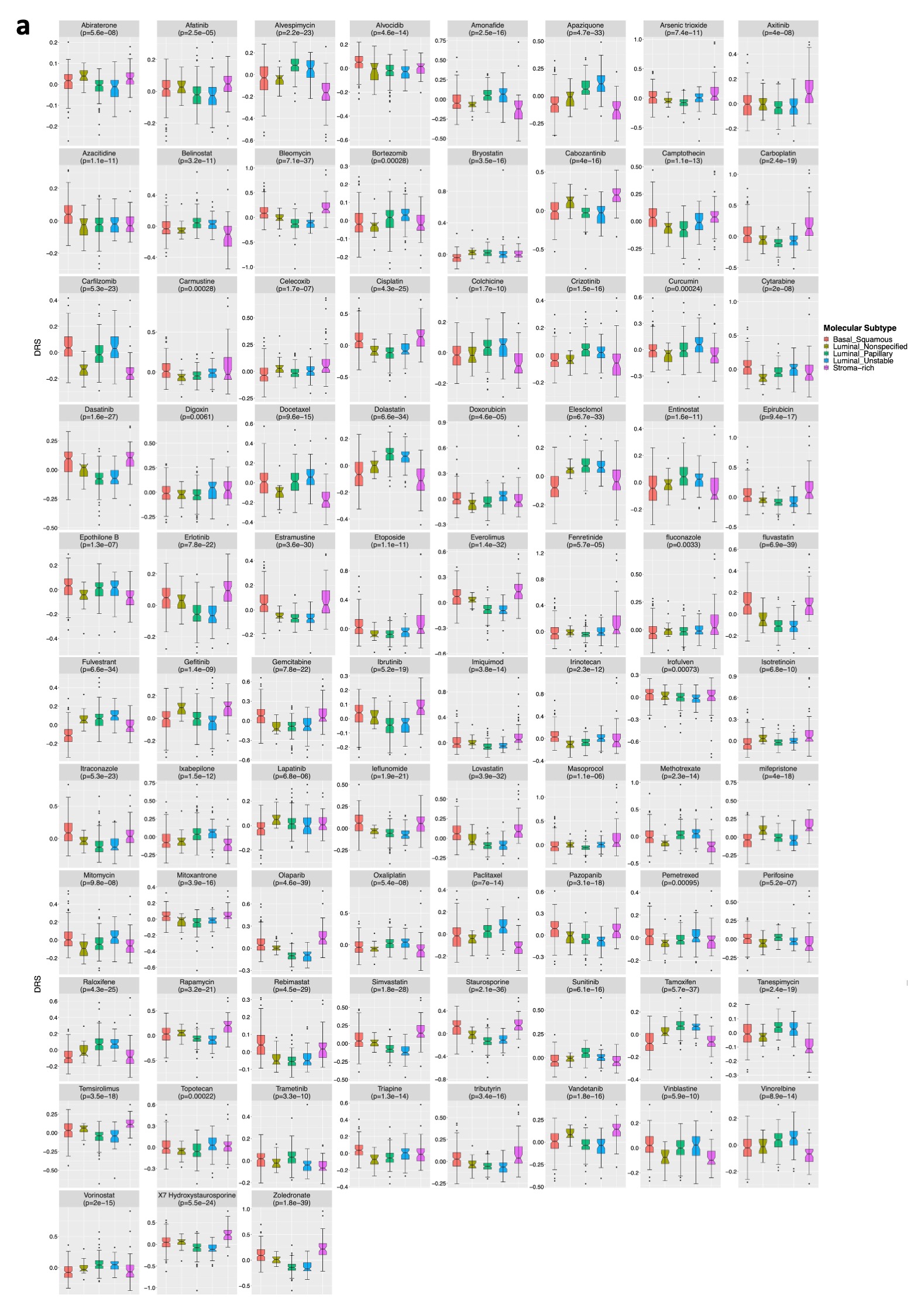
**

**
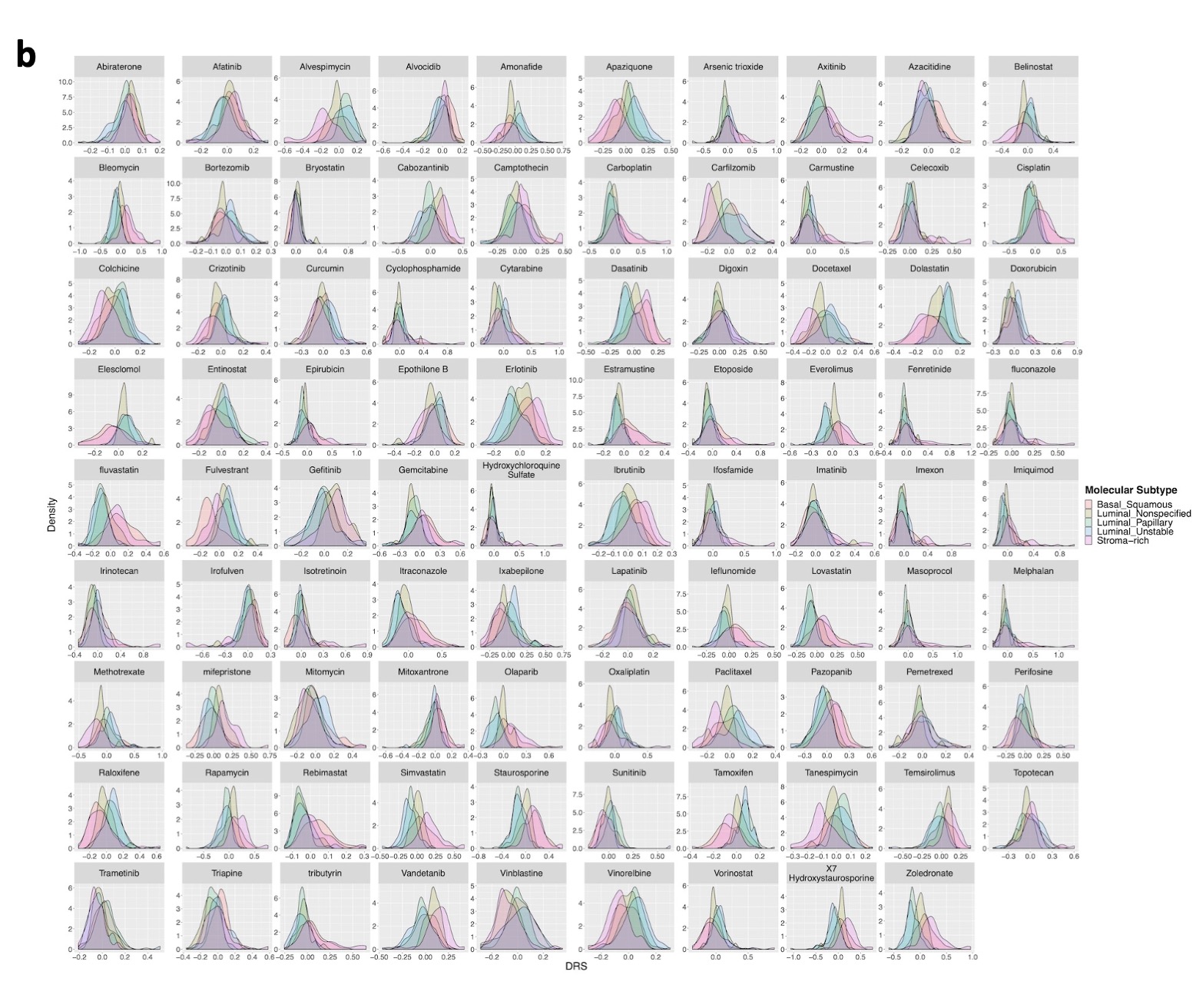
**

**Suppl. Fig. 8 a, b:** A Kruskal-Wallis rank sum test was used to compare the DRS scores across the molecular subtypes independently for each available drug. Note that samples without a subtype definition as well as those in the NE-like subtype were excluded from this analysis due to the low sample number. In (**a**) the box-plots illustrate 83 drugs (of a total of 89 drugs with available DRS information) with significant prediction differences among molecular subtypes (Kamoun et al., European Urology, 2020). (**b**) Density plots for all 89 drugs with available DRS information depict the distribution density of the DRS per molecular subtype. (R Core Team (2019). R: A language and environment for statistical computing. R Foundation for Statistical Computing, Vienna, Austria. URL: <https://www.R-project.org/>) ^57^
